# Influence of Ranibizumab versus laser photocoagulation on radiation retinopathy (RadiRet) - a prospective randomized controlled trial

**DOI:** 10.1007/s00417-020-04618-7

**Published:** 2020-02-28

**Authors:** Ira Seibel, Daniela Vollhardt, Aline I. Riechardt, Matus Rehak, Sabine Schmied, Petra Schiller, Oliver Zeitz, Martin Hellmich, Antonia M. Joussen

**Affiliations:** 1 Department of Ophthalmology, Charité University Medicine Berlin, Hindenburgdamm 30, 12200 Berlin, Germany; 2 Clinical Trial Centre Cologne (CTCC), University of Cologne, Gleueler Str. 269, 50935 Cologne, Germany; 3 Institute of Medical Statistics and Computational Biology (IMSB), University of Cologne, Bachemer Str. 86, 50931 Cologne, Germany

**Keywords:** Radiation retinopathy, Laser photocoagulation, Ranibizumab, Intravitreal therapy, Uveal melanoma

## Abstract

**Purpose:**

To demonstrate superiority of intravitreal ranibizumab 0.5 mg compared to focal and peripheral laser treatment in patients with radiation retinopathy for choroidal melanoma.

**Methods:**

Inclusion criteria were as follows: patients with radiation retinopathy and visual acuity impairment due to radiation maculopathy accessible for laser therapy, age ≥ 18 years, and BCVA less than 20/32. The main objective was to study the change in best-corrected visual acuity (BCVA) over 6 months from ranibizumab 0.5 mg (experimental) compared to focal laser of the macula and panretinal laser treatment of the ischemic retina (control) in patients with radiation retinopathy in choroidal melanoma. The secondary objectives of the radiation retinopathy study were to compare functional and anatomical results between ranibizumab and laser group over 12 months and to measure the frequency of vitreous hemorrhage and rubeosis iridis.

**Results:**

The intention-to-treat analysis included 31 patients assigned to ranibizumab (*n* = 15) or laser treatment (*n* = 16). In terms of BCVA at month 6, ranibizumab was superior to laser treatment, with an advantage of 0.14 logMAR, 95% CI 0.01 to 0.25, *p* = 0.030. The positive effect of ranibizumab disappeared after treatment was discontinued. Similar results without statistically significant difference were found with respect to macular thickness. In both groups, no change was observed at month 6 in the size of ischemia in the macula or periphery compared to baseline. There was 1 case of vitreous hemorrhage in the laser group and no case of rubeosis iridis over time.

**Conclusions:**

This study showed a statistically significant improvement in visual acuity and clear superiority of ranibizumab compared to laser treatment up to 26 weeks, but this effect disappeared at week 52 after completion of intravitreal treatment. Ranibizumab and PRP are considered equivalent in terms of the non-appearance of proliferative radiation retinopathy during the study.

**Trial registration:**

EudraCT Number: 2011-004463-69

**Electronic supplementary material:**

The online version of this article (10.1007/s00417-020-04618-7) contains supplementary material, which is available to authorized users.

## Introduction

Radiation retinopathy is an ischemic retinopathy caused by irradiation damage to the retina and choroid. In practice, radiation retinopathy is a common complication following a radiotherapy for intraocular tumors with radiation maculopathy being the leading cause of irreversible vision loss in patients treated for uveal melanoma. Intravitreal injections of anti-VEGF or corticosteroids have been shown to maintain or improve the visual acuity and reduce cystoid macular edema when administered over a long period of time [1–4].

The clinical appearance of radiation retinopathy mimics some important features of diabetic retinopathy such as exudates, hemorrhages, cotton wool spots, capillary non-perfusion, and the occurrence of macular edema [5–7].

Due to the clinical and pathophysiological similarities, learnings and techniques from diabetic retinopathy may be transferred. Panretinal laser photocoagulation (PRP) of the ischemic retina is useful in the prophylaxis of proliferative diabetic retinopathy and may stabilize macular edema when intravitreal injections are discontinued [8, 9].

Whether PRP leads to the same effect in radiation retinopathy has not yet been proven in studies.

The aim of the radiation retinopathy (RadiRet) study was to address this gap. Therefore, the RadiRet study was designed to compare monthly ranibizumab injections for a maximum interval of 6 months with focal and PRP in patients diagnosed with radiation retinopathy.

The RadiRet study is hereby the first randomized controlled trial comparing ranibizumab with laser treatment in radiation retinopathy.

## Methods

**Fig. 1:**
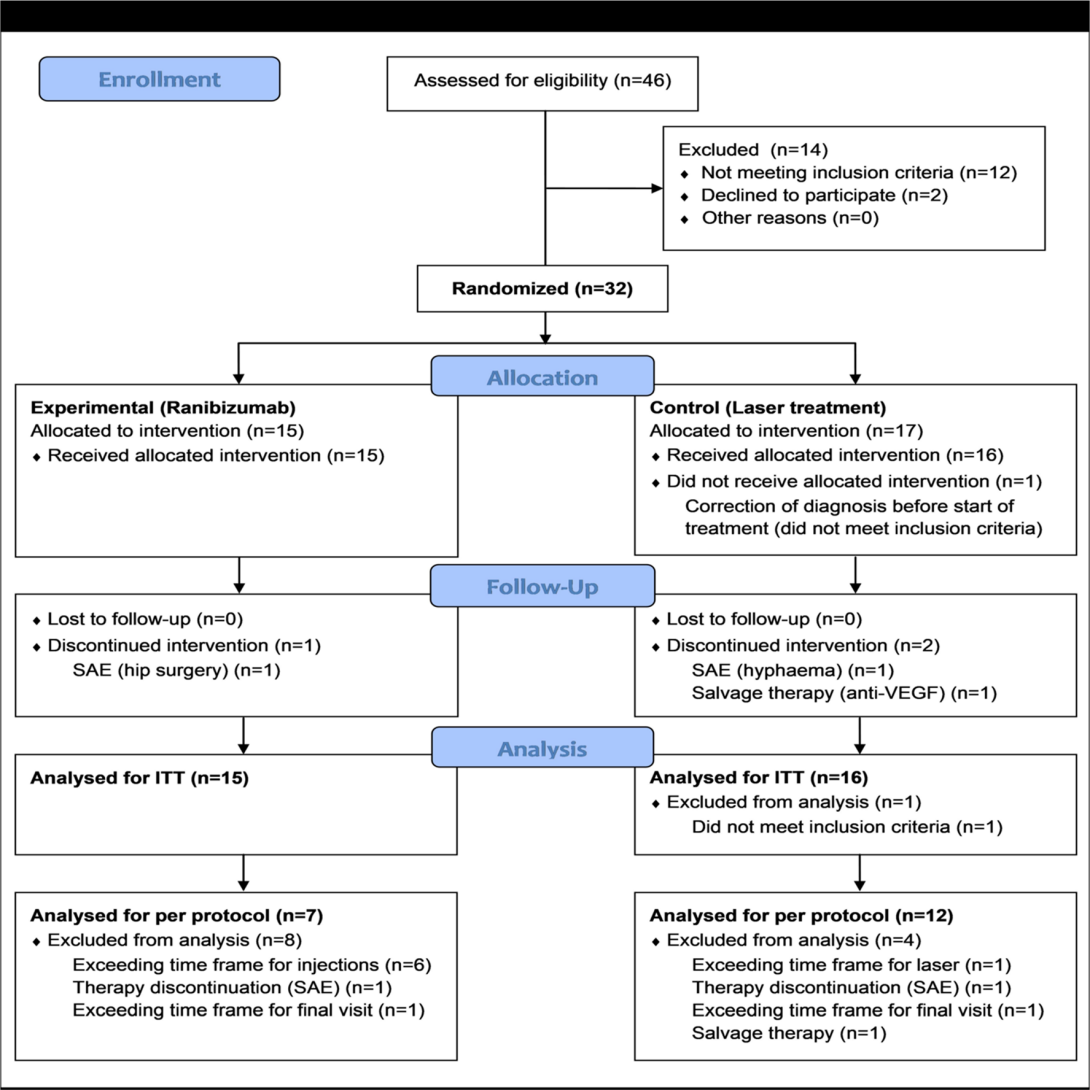
The CONSORT flow diagram shows the trial profile of RadiRet. In total, 32 patients were randomized. The full analysis set (FAS) was defined as all trial subjects enrolled into the trial and randomized. One patient (Arm L) was randomized but did not receive the allocated intervention because the diagnosis was corrected before start of treatment and inclusion criteria were not met. Therefore, 31 patients were analyzed for FAS (*n* = 15 (Arm R); *n* = 16 (Arm L)). Analysis of the FAS was done according to the intention-to-treat principle, that is, all patients were evaluated for the group to which they have been assigned

The RadiRet study was a therapeutic-exploratory, two-arm, randomized, parallel group, single-masked, active-controlled phase II clinical trial with a follow-up period of 12 months (Fig. 1). It has been registered in the EU Clinical Trials Register, EudraCT Number: 2011-004463-69. Institutional Review Board (IRB)/Ethics Committee approval was obtained. All procedures performed in the study were in accordance with the ethical standards of the institutional research committee and with the 1996 Helsinki declaration. The trial protocol and any amendments were prepared in accordance with the Declaration of Helsinki in the version of October 1996 (48th General Assembly of the World Medical Association, Somerset West, Republic of South Africa). Written informed consent was obtained from all individual participants included in the study.

The primary objective was to investigate the change from baseline in best-corrected visual acuity (BCVA) over 6 months of ranibizumab 0.5 mg (experimental) in comparison to focal laser treatment of the macula and laser treatment of ischemic retina (control) in patients with radiation retinopathy secondary to radiation therapy of uveal melanoma.

The secondary objectives of the RadiRet study were to compare functional and anatomic outcomes between ranibizumab and laser groups over 12 months. The proportion of patients with changes in BCVA after 6 and 12 months and the rate of peripheral ischemia and vitreous hemorrhages were analyzed. As third objective, safety of 0.5 mg ranibizumab was investigated.

Safety endpoints further included local and systemic tumor control, investigation for key arterial thromboembolic events, death, and non-ocular hemorrhage; ocular endpoints comprised intraocular pressure, endophthalmitis, rhegmatogenous retinal detachment, retinal tear, vitreous hemorrhage, lens damage, and signs of ocular inflammation.

The RadiRet study included patients with retinopathy due to radiation of uveal melanoma that presented with radiation maculopathy (visual impairment due to focal or diffuse ME in the irradiated eye that was eligible for laser treatment) and clinical signs of radiation retinopathy, i.e., cotton wool spots, hemorrhages, vascular ischemia. All patients were ≥ 18 years of age, and BCVA was less than 20/32 at the primary visit.

Exclusion criteria were participation in other interventional trials and concomitant conditions in the study eye which in the opinion of the investigator could prevent BCVA improvement, e.g., tumor recurrence, tumor growth underneath the macula, tumor endoresection, and/or previous vitrectomy. Patients with proliferative retinopathies or macular edema due to reasons other than irradiation, e.g., diabetic retinopathy, vein occlusion, or Irvine-Gass syndrome, were excluded as were patients with previous treatment with anti-angiogenic drugs or intravitreal corticosteroids or any other investigational drug within 3 months prior to randomization, or prior laser photocoagulation treatment within 3 months (focal/grid laser) or 6 months (panretinal) prior to study entry. Furthermore, patients with known hypersensitivity against local anesthetics or iodine, patients with anamnestically confirmed stroke or preliminary stages of stroke, or patients with history of myocardial infarction, pregnant or nursing women, and failure to use highly effective contraceptive methods were excluded from the trial.

Assuming a within-group standard deviation of 8.5 letters (calculated based on Finger et al. [1]), a sample size of 27 patients per treatment arm was considered sufficient to detect a clinically relevant difference of 5 letters in the primary variable at a one-sided level of 10% with 80% power (*δ*/*σ* ≈ 0.6). Randomization was computer-generated and based on permuted blocks of varying length and stratified by radiation dose to macula and disc. In order to account for stratification and attrition, 27/0.9 = 30 patients were planned to be randomly assigned per treatment arm. Assuming an effect size of 0.6, at least 20 evaluable patients are required to observe an effect in the hypothesized direction with 90% probability. Given the orphan nature of disease, enrollment went expectedly slow. Therefore, and to limit the number of subjects exposed to the trial, the decision was taken to perform a premature final analysis after approximately 50% of the originally planned enrollment target was achieved. At that time, 31 patients were enrolled. This was justified as only 20 evaluable patients are sufficient to observe a treatment effect in the hypothesized direction with a probability of at least 90% (given the expected effect size of 0.6). The study protocol was amended accordingly. The amendment was approved by the local Ethics Committee and competent authorities.

Each monthly visit included refraction, BCVA testing using ETDRS charts, intraocular pressure (IOP), slit lamp examination, and OCT assessment of macular thickness and anatomy. Fundus photography and fluorescein angiography using the Heidelberg retina angiograph [Heidelberg Engineering] were performed at the initial visit and at month 6.

### Ranibizumab group versus laser group

At baseline, patients were randomized to receive either ranibizumab intravitreal injections or laser photocoagulation. Patients returned monthly for visits. At the first three visits (baseline, month 1 (week 4), and month 2 (week 8)), ranibizumab injections were mandatory for the ranibizumab group. During further visits up to month 6 (week 26), patients were able to receive additional injections according to the pro re nata regimen if one or more of the following criteria were met: (1) visual acuity dropped by > 5 letters from best value observed during treatment (including baseline); (2) evidence of macula edema as determined by optical coherence tomography (OCT); (3) presence of optic disc edema as determined by fundoscopy. Injection was discontinued when no further BCVA improvement due to treatment at 2 previous consecutive visits was seen, or BCVA was ≥ 84 letters at the last 2 consecutive visits.

Laser treatment of the macula and periphery served as comparator treatment and was performed by Visulas 532s laser device of Carl Zeiss Meditec AG, Jena Germany. Due to the inclusion criterion of macular edema, which had to be eligible for focal laser, focal laser photocoagulation was obligatory. PRP was performed only if peripheral ischemia was observed in fluorescein angiographies at baseline. Treatment was performed as focal treatment according to the ETDRS laser protocols for diabetic macular edema (modified grid technique in areas of edema as evidenced by OCT using 50 μm burns sparing the fovea) and as disseminated coagulation in ischemic areas in the periphery (200 μm burns were placed 2 burn diameters apart) [10]. Laser retreatment was allowed if time elapsed since last laser treatment was at least 3 months and in addition one or more of the following criteria were met: (1) visual acuity drops by > 5 letters from best observed on treatment (including baseline); (2) evidence of ischemic areas on fluorescein angiographies; (3) macular edema as evidenced by OCT; or (4) optic disc edema present on funduscopy.

Both groups were eligible to receive treatments according to their treatment assignment up to week 26, when the primary endpoint was assessed. During the follow-up period from week 26 to week 52, no study treatments were administered. At each visit, a full clinical examination was carried out including visual acuity assessment (BCVA) using ETDRS charts. In order to minimize detection bias, the BCVA examiner, imaging operator, and readers of images were masked to treatment assignment. BCVA and macular thickness were analyzed in a masked fashion.

### Statistical analysis

The analysis was according to the intention-to-treat, i.e., including all patients enrolled into the trial, randomized and treated (full analysis set, FAS).

The change from baseline in BCVA and macular thickness over time was evaluated by analysis of covariance (ANCOVA) with main effects for baseline, treatment, and dose to macula and disc (type II sums of square). The interaction treatment × dose was explored in a sensitivity analysis. Outcome measures were windowed according to ranges defined in the study protocol; any missing values were imputed by the last observation carried forward (LOCF). Complementary, a linear mixed model for repeated measures over time was fitted with ARH(1)-structured variance-covariance matrix (heterogenous first-order autoregressive) and pairwise contrasts of estimated marginal means (EMMs). Further inferential statistics were calculated for descriptive purpose only; thus, no adjustment for multiple testing was applied.

Quantitative data were summarized by mean, standard deviations or median, and interquartile range (IQR), contingent on distributional characteristics; qualitative data by count (percentage). Subgroup analyses were done by gender and, partly, dose to macula and disc. Statistical calculations were done with SPSS Statistics 25 (IBM Corp., Armonk, NY, USA).

## Results

From 10/2013 to 12/2015, 46 patients were screened, thirty-two of which could be randomized. (The study was prematurely terminated after 31 included patients.)

All 15 patients who were randomized to receive ranibizumab started treatment. Out of 17 patients who were randomized to the laser group, only 16 were treated. One patient was excluded from the FAS before administration of first treatment as one inclusion criterion was not fulfilled (diagnosis of radiation retinopathy had to be revised).

Patients randomized to the ranibizumab group received a median of 5 injections (range 4 to 6). All laser group patients received 1 focal treatment. For 10 out of the 16 laser group patients, one additional peripheral laser treatment was applied.

Baseline characteristics are depicted in Table 1. None of the patients presented with rubeosis iridis at baseline.

**Table 1:** Demographics, baseline characteristics

	Total (*n* = 31)	Ranibizumab (*n* = 15)	Laser (*n* = 16)
Median age (IQR) (years)	67 (56–75)	73 (65–78)	62 (53–76)
Gender			
Female	6 (19%)	2 (13%)	4 (25%)
Male	25 (81%)	13 (87%)	12 (75%)
Study eye			
Right eye	18 (58%)	10 (67%)	8 (50%)
Left eye	13 (42%)	5 (33%)	8 (50%)
Radiation dose to macula and disc			
≤ 40 Gy	20 (65%)	10 (67%)	10 (63%)
> 40 Gy	11 (36%)	5 (33%)	6 (38%)
Largest tumor diameter (mm)	10.5 (4)	10.1 (4)	10.9 (3)
Maximal tumor height (mm)	3.9 (2)	3.4 (2)	4.3 (2)
Localization optic disc	6 (19%)	2 (13%)	4 (25%)
Localization fovea	4 (13%)	2 (13%)	2 (13%)
Localization ciliary body	3 (10%)	0 (0%)	3 (19%)
Median tumor distance to optic disc (IQR) (mm)	3.5 (0.5–6.0)	4.7 (0.5–7.5)	3.4 (0.4–5.9)
Median tumor distance to fovea (IQR) (mm)	3.2 (2.3–3.7)	3.4 (3.2–4.6)	2.7 (1.5–2.8)
Kind of radiation			
Proton	26 (84%)	13 (87%)	13 (81%)
Ruthenium applicator	5 (16%)	2 (13%)	3 (19%)
Diabetes	4 (13%)	3 (20%)	1 (6%)
Hypertension	18 (58%)	11 (73%)	7 (44%)
Other disease	25 (81%)	13 (87%)	12 (75%)

Data are *n* (%) or mean (SD) unless stated otherwise

*IQR* interquartile range

### Efficacy

#### Primary endpoint

**Fig. 2:**
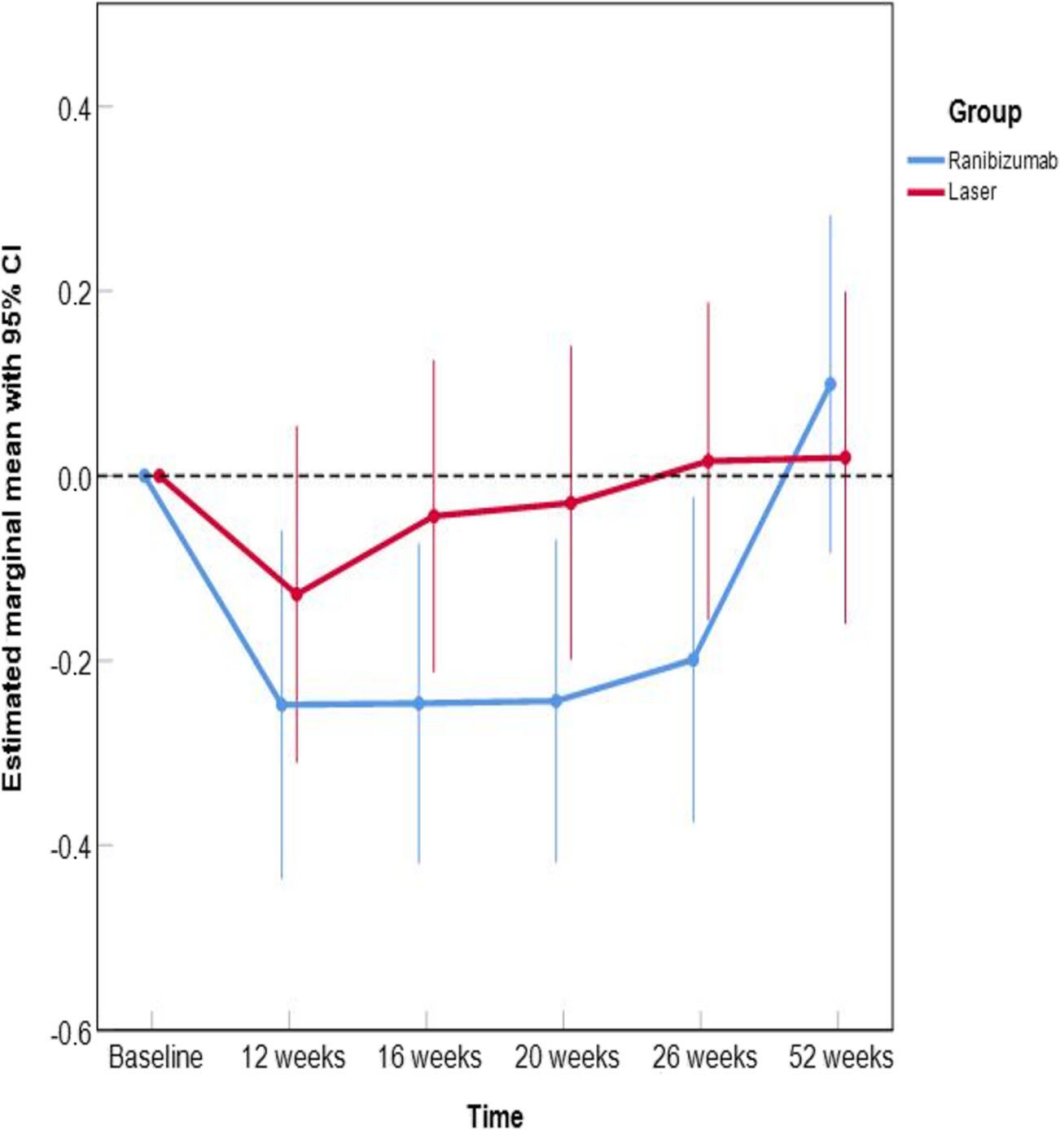
Change in BCVA (logMAR) from baseline, the mixed model approach shows significant group differences at weeks 20 (0.203, 95% CI (0.002 to 0.404), *p* = 0.048) and 26 (0.208, 95% CI (0.004 to 0.413), *p* = 0.046)

The average change in BCVA from baseline over 26 weeks’ treatment with ranibizumab was − 0.16 logMAR, 95% CI (− 0.25 to − 0.08) versus 0.03, 95% CI (− 0.12 to 0.05) with laser treatment. This translates into a statistically significant advantage for ranibizumab of 0.14 logMAR (equivalent to approximately 7 ETDRS chart letters), 95% CI 0.01 to 0.25, *p* = 0.030 (see Fig. 2, Table 2). The mixed model approach showed significant group differences at weeks 20 (0.203, 95% CI (0.002 to 0.404), *p* = 0.048) and 26 (0.208, 95% CI (0.004 to 0.413), *p* = 0.046).

**Table 2:** Key outcome measures

		Mean ± SD, *n*	Mean ± SD, *n*	Mean ± SD, *n*	ANCOVA (LOCF), EMM, 95% CI	
	Arm	Baseline	26 weeks	52 weeks	Average change (AUC) over 26 weeks	Average change (AUC) over 52 weeks
BCVA (logMAR)						
Overall						
	Ranibizumab	0.59 ± 0.31, 15	0.38 ± 0.33, 13	0.75 ± 0.51, 11	− 0.16, − 0.25 to − 0.08	− 0.11, − 0.24 to 0.02
	Laser	0.71 ± 0.45, 16	0.74 ± 0.51, 13	0.82 ± 0.47, 11	− 0.03, − 0.12 to 0.05	0.00, − 0.12 to 0.13
	Contrast, 95% CI, p				0.14, 0.01 to 0.25, 0.030	0.11, − 0.06 to 0.29, 0.195
Dose to macula and disc					0.625 (interaction)	0.212 (interaction)
≤ 40 Gy	Ranibizumab	0.50 ± 0.24, 10	0.35 ± 0.26, 9	0.70 ± 0.42, 8	− 0.17, − 0.28 to − 0.05	− 0.10, − 0.23 to 0.04
	Laser	0.68 ± 0.46, 10	0.60 ± 0.49, 9	0.71 ± 0.55, 7	− 0.06, − 0.17 to 0.06	− 0.08, − 0.21 to 0.06
	Contrast, 95% CI, *p*				0.11, − 0.06 to 0.27, 0.182	0.02, − 0.17 to 0.21, 0.823
> 40 Gy	Ranibizumab	0.79 ± 0.36, 5	0.46 ± 0.50, 4	0.89 ± 0.81, 3	− 0.18, − 0.34 to − 0.02	− 0.16, − 0.47 to 0.14
	Laser	0.78 ± 0.47, 6	1.06 ± 0.45, 4	1.01 ± 0.21, 4	− 0.01, − 0.16 to 0.14	0.09, − 0.19 to 0.37
	Contrast, 95% CI, *p*				0.17, − 0.05 to 0.39, 0.110	0.26, − 0.16 to 0.67, 0.193
Central foveal thickness						
Overall						
	Ranibizumab	478 ± 153, 15	387 ± 146, 13	509 ± 193, 10	− 73.5, − 115.2 to − 31.8	− 61.0, − 111.0 to − 11.0
	Laser	509 ± 149, 16	452 ± 159, 12	460 ± 179, 11	− 22.2, − 62.2 to 17.8	− 28.1, − 76.0 to 19.8
	Contrast, 95% CI, *p*				51.3, − 5.4 to 108.0, 0.074	32.9, − 35.0 to 100.8, 0.329
Dose to macula and disc					0.233 (interaction)	0.697 (interaction)
≤ 40 Gy	Ranibizumab	464 ± 164, 10	397 ± 143, 9	552 ± 193, 8	− 75.9, − 109.6 to − 42.2	− 44.1, − 83.0 to − 5.3
	Laser	508 ± 134, 10	499 ± 149, 9	463 ± 146, 7	− 1.7, − 35.4 to 32.0	− 5.9, − 44.8 to 32.9
	Contrast, 95% CI, *p*				74.2, 26.3 to 122.1, 0.005	38.2, − 17.1 to 93.4, 0.163
> 40 Gy	Ranibizumab	507 ± 141, 5	365 ± 172, 4	339 ± 46, 2	− 60.3, − 177.9 to 57.3	− 73.4, − 217.6 to 70.9
	Laser	512 ± 185, 6	310 ± 106, 3	454 ± 254, 4	− 53.6, − 161.0 to 53.7	− 57.5, − 189.2 to 74.1
	Contrast, 95% CI, *p*				6.7, − 152.6 to 165.9, 0.925	15.8, − 179.5 to 211.1, 0.857
BCVA (logMAR)						
Overall						
	Ranibizumab	0.59 ± 0.31, 15	0.38 ± 0.33, 13	0.75 ± 0.51, 11	− 0.16, − 0.25 to − 0.08	− 0.11, − 0.24 to 0.02
	Laser	0.71 ± 0.45, 16	0.74 ± 0.51, 13	0.82 ± 0.47, 11	− 0.03, − 0.12 to 0.05	0.00, − 0.12 to 0.13
	Contrast, 95% CI, *p*				0.14, 0.01 to 0.25, 0.030	0.11, − 0.06 to 0.29, 0.195
Central foveal thickness						
Overall						
	Ranibizumab	478 ± 153, 15	387 ± 146, 13	509 ± 193, 10	− 73.5, − 115.2 to − 31.8	− 61.0, − 111.0 to − 11.0
	Laser	509 ± 149, 16	452 ± 159, 12	460 ± 179, 11	− 22.2, − 62.2 to 17.8	− 28.1, − 76.0 to 19.8
	Contrast, 95% CI, *p*				51.3, − 5.4 to 108.0, 0.074	32.9, − 35.0 to 100.8, 0.329

*ANCOVA* analysis of covariance, *AUC* area under curve, *BCVA* best-corrected visual acuity, *CI* confidence interval, *EMM* estimated marginal mean, *n* number of subjects, *SD* standard deviation, *LOCF* last observation carried forward

#### Secondary endpoints

The positive effect of ranibizumab on BCVA vanished following week 26, i.e., after treatment was stopped.

The average change from baseline in BCVA over 52 weeks was 0.11 logMAR, 95% CI − 0.06 to 0.29, *p* = 0.195. At week 26, *n* = 11 patients of 13 (i.e., 85%, 95% CI 55 to 98) showed an improvement in BCVA from baseline on ranibizumab versus 9 of 13 (i.e., 69%, 95% CI 39 to 91) on laser treatment; at week 52, there were 6 of 11 (i.e., 55%, 95% CI 23 to 83) versus 5 of 11 (i.e., 46%, 95% CI 17 to 77).

**Fig. 3:**
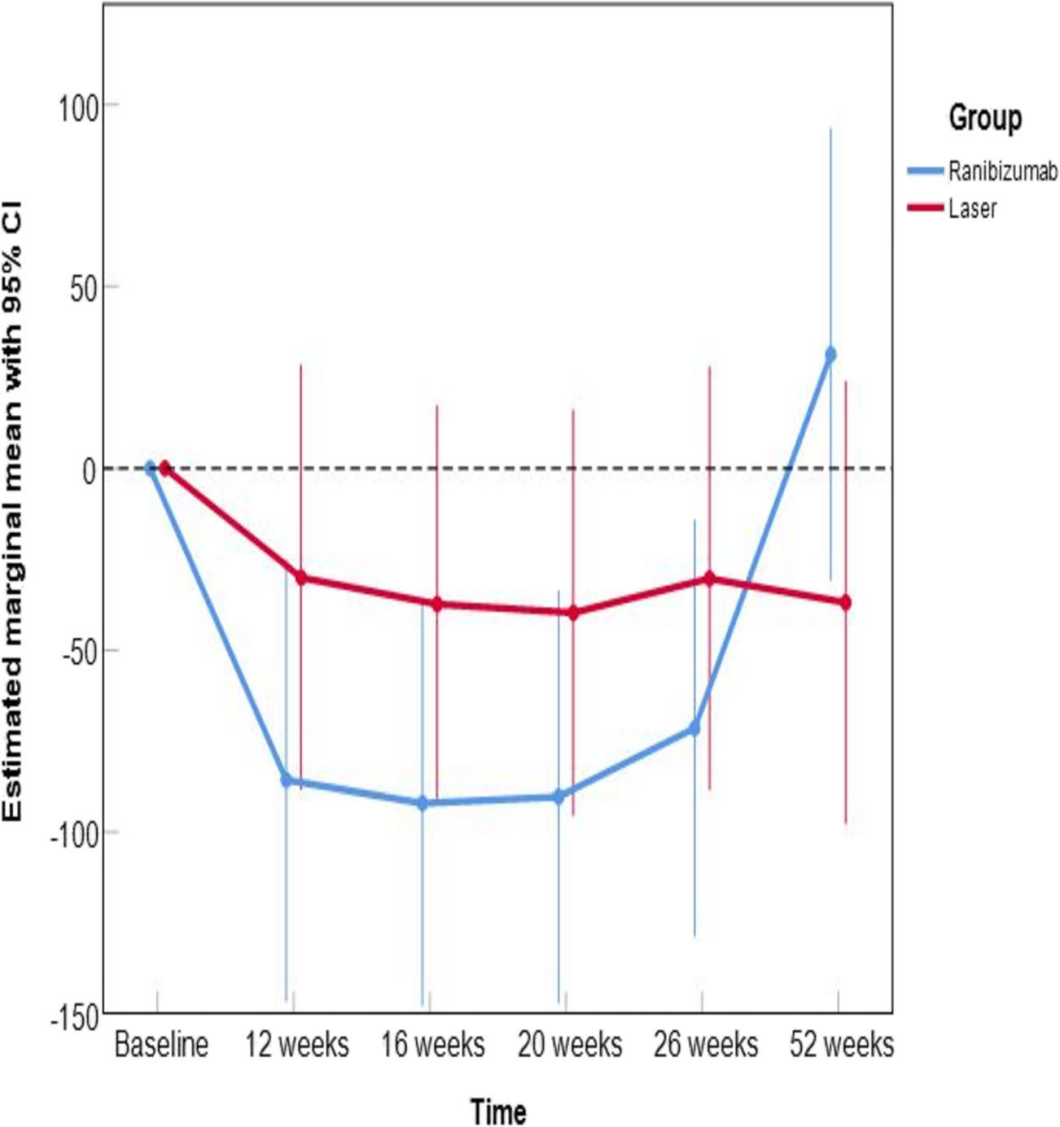
Change in foveal thickness (μm) from baseline, the mixed model approach shows similar group differences as for BCVA however not statistically significant. For example, at week 26, 41.3, 95% CI − 40.5 to 123.0, *p* = 0.31

Regarding the average change in central foveal thickness from baseline over 26 weeks, treatment with ranibizumab differed by 51.3 μm, 95% CI (− 5.4 to 108.0), *p* = 0.074, from laser treatment (see Table 2). This difference in foveal thickness vanished following week 26, i.e., after treatment was stopped, while the laser group remained stable (see Fig. 3). Over 52 weeks, the difference was 32.9 μm, 95% CI − 35.0 to 100.8, *p* = 0.329. The mixed model approach shows similar group differences as for BCVA; however, these were not statistically significant. For example, at week 26, 41.3, 95% CI − 40.5 to 123.0, *p* = 0.318.

In both groups, no change was observed in the size of capillary dropout areas in the macular or periphery comparing baseline to month 6.

There was 1 case of vitreous hemorrhage in the laser group due to posterior vitreous detachment and no case with rubeosis iridis over time.

#### Subgroup analyses

Low irradiation doses (< 40 Gy) compared to higher doses (> 40 Gy) did not interact with the study medication and had no effect on primary and secondary endpoints, *p* = 0.405 (Table 2).

### Safety

There were no local recurrences during the entire study period. In total, 16 serious adverse events (SAEs) occurred in 11 patients. Hereof, 7 SAEs occurred in 7 patients in the ranibizumab group and 9 SAEs occurred in 4 patients in the laser group (see Table 3). There was no difference between the groups regarding the ocular safety measures of which none was reported, such as intraocular pressure changes, endophthalmitis, rhegmatogenous retinal detachment, retinal tear, or vitreous hemorrhage. None of the reported SAEs was life threatening, resulted in death, or a change in dose. A trend towards more gastrointestinal disorders in the ranibizumab group (i.e., 5 AEs in 5 patients versus 1 in 1, *p* = 0.083) might be visible however not drug related.

**Table 3:** Incidence of (serious) adverse events

System organ class, preferred term (both MedDRA)	Total (*n* = 31)		Ranibizumab (*n* = 15)		Laser (*n* = 16)	
	Patients (%)	Events	Patients (%)	Events	Patients (%)	Events
Any AE	31 (100)	118	15 (100)	64	16 (100)	54
Cardiac disorders	1 (3)	1	1 (7)	1	0	0
Ear and labyrinth disorders	1 (3)	1	0	0	1 (6)	1
Eye disorders	23 (74)	69	12 (80)	36	11 (69)	33
Gastrointestinal disorders	6 (19)	6	5 (33)	5	1 (6)	1
General disorders and administration site	2 (6)	2	1 (7)	1	1 (6)	1
Infections and infestations	11 (36)	11	4 (27)	4	7 (44)	7
Investigation	1 (3)	1	0	0	1 (6)	1
Metabolism and nutrition disorders	1 (3)	1	1 (7)	1	0	0
Musculoskeletal and connective tissue disorders	7 (23)	11	2 (13)	5	5 (31)	6
Neoplasms benign, malignant, and unspecified (incl cysts and polyps)	3 (10)	3	2 (13)	2	1 (7)	1
Nervous system disorders	3 (10)	3	2 (13)	2	1 (7)	1
Psychiatric disorders	1 (3)	1	0	0	1 (7)	1
Reproductive system and breast disorders	1 (3)	1	1 (7)	1	0	0
Respiratory, thoracic, and mediastinal disorders	1 (3)	1	1 (7)	1	0	0
Vascular disorders	3 (10)	4	2 (13)	3	1 (7)	1
Any SAE	11 (31)	16	7 (47)	7	4 (11)	9
Eye disorders						
Cataract	1 (3)	1	1 (7)	1	0	0
Subconjunctival bleeding	1 (3)	2	0	0	1 (6)	1
Hyphema	1 (3)	3	0	0	1 (6)	3
Macular ischemia	1 (3)	1	1 (7)	1	0	0
Radiation retinopathy	1 (3)	1	0	0	1 (6)	1
Vitreous hemorrhage	1 (3)	1	0	0	1 (6)	1
Gastrointestinal disorders						
Umbilical hernia	1 (3)	1	1 (7)	1	0	0
Infections and infestations						
Furuncle	1 (3)	1	0	0	1 (6)	1
Injury, poisoning, and procedural complications						
Incisional hernia	1 (3)	1	1 (7)	1	0	0
Musculoskeletal and connective tissue disorders						
Osteoarthritis	2 (6)	2	1 (7)	1	1 (6)	1
Neoplasms benign, malignant, and unspecified (incl cysts and polyps)						
Metastasis to liver	1 (3)	1	1 (7)	1	0	0
Prostate cancer	1 (3)	1	0	0	1 (6)	1
Nervous system disorders						
Cerebral infarction	1 (3)	1	1 (7)	1	0	0

*MedDRA* Medical Dictionary for Regulatory Activities, *AE* adverse event, *SAE* serious adverse event

## Discussion

This randomized controlled trial compared the efficacy and safety of intravitreal ranibizumab treatment with laser treatment in radiation retinopathy secondary to radiation of uveal melanoma.

Ranibizumab-treated patients had significantly better BCVA and showed rapid regression of macular edema. On the other hand, the study also showed that the superior efficacy of ranibizumab is contingent to continuous treatment as after cessation of therapy post week 26, visual acuity decreased and central retinal thickness increased back to baseline.

As in this RadiRet study, it has been shown that determining the right treatment frequency with anti-VEGF injections is a key success factor for maintaining the favorable treatment results [1, 2, 11].

Importantly, the RadiRet study also contributes systematic safety information in a comparative setting. The safety profile of ranibizumab in radiation retinopathy suggests safe use, as reported in other studies [1, 11–13].

Of importance, we did not see any progression of macular ischemia in fluorescein angiography independent of the treatment group during the course of the study. Several studies had raised the question of anti-VEGF-induced macular ischemia progression. However, due to lack of information from fluorescein angiographies in these studies, a macular ischemia in the absence of visual improvement could not be excluded [2, 12].

Other previous studies with VEGF inhibition in radiation retinopathy focussed on radiation maculopathy prevention [11, 13]. These studies demonstrated that the occurrence of radiation maculopathy can be reduced from 50–68% to 33–40% by preventive injections over 24 months.

If radiation maculopathy nevertheless occurs, therapeutic injections become necessary—the topic of the RadiRet study.

RadiRet’s objective was to demonstrate the superiority of intravitreal ranibizumab therapy over central focal laser in terms of visual acuity over 6 months. The termination of the injection therapy after 6 months was intended to demonstrate the effect of anti-VEGF therapy on peripheral ischemia and its complications including proliferation and hemorrhages. In this study, none of the patients in the anti-VEGF group developed bleeding or neovascularization compared to the laser group in which there was a case of bleeding due to posterior vitreous detachment (without statistical significant difference).

Even if the groups appear to be equal with regard to these points, no conclusion should be drawn due to the short follow-up period.

In general, the incidence of proliferative radiation retinopathy described in the literature is very low, especially given the small size of irradiated tumors included into this study [14, 15].

RadiRet study included only a limited number of patients. In view of the rare disease and in addition to the exclusion criterion of a previous vitrectomy, which is routinely performed due to genetic examination of the tumor, enrolment progressed expectedly slowly. Preceding vitrectomy might alter the quantity of intraocular VEGF and improve oxygen diffusion [16, 17] and was therefore excluded from the study.

The RadiRet study employed a pro re nata (PRN) treatment scheme for ranibizumab. As all patients required ranibizumab re-treatments (4–6 injections) after the initiation with three monthly injections, it could be conceivable that results would have been even better with fixed continuous monthly treatment or treat-and-extend regimen.

Despite the small study population, the RadiRet study was able to demonstrate a promising effect. Thus, a continuation of the study would have entailed ethical conflict potential. It remains to be elucidated how long continuation of anti-VEGF treatment remains to be functionally superior over laser treatment.

RadiRet compared the ranibizumab treatment to laser photocoagulation. Since RadiRet showed that ranibizumab was significantly superior to focal laser up to 6 months, a third, combined group with laser treatment of peripheral ischemia and treatment of macular edema with ranibizumab would be interesting to identify differences in injection frequency and long-term visual outcome.

In conclusion, BCVA was clearly superior after ranibizumab compared to focal and peripheral laser treatment as long as the injections continued. After termination of treatment, there was no BCVA difference between ranibizumab and laser therapy. Since we did not see a development into proliferative radiation retinopathy in any of the groups during the study period, ranibizumab and PRP are considered equivalent in this respect.

Intravitreal injections are the therapy standard to achieve visual acuity improvement or stabilization in patients with radiation maculopathy. While intravitreal injections require good patient adherence, PRP is completed after 1–2 treatments. In future studies, photocoagulation of peripheral ischemia should be investigated in addition to intravitreal injections.

## Electronic supplementary material

ESM 1 (PDF 523 kb)

ESM 2 (DOCX 19 kb)
