# Supplementary material for: Influence of Ranibizumab versus laser photocoagulation on radiation retinopathy (RadiRet) - a prospective randomized controlled trial
Source: Graefes Arch Clin Exp Ophthalmol. 2020 Feb 28;258(4):869–78. doi: 10.1007/s00417-020-04618-7 (PMC7575494; doi:10.1007/s00417-020-04618-7)
Supplement: Supplementary file 1 — (PDF 523 kb) [file 417_2020_4618_MOESM1_ESM.pdf]

CHARITÉ

ChariteCentrum für Audiologie / Phoniatrie, Augen und HNO-Heilkunde

Universität zu Köln

TRIAL PROTOCOL

Investigation of Radiation Retinopathy (Radi-Ret Study)

Subtitle: Influence of Lucentis® on radiation retinopathy after irradiation of choroidal melanoma

Sponsor

Charité – University Medicine Berlin

Schumannstr. 20/21

10117 Berlin

Germany

Principal Coordinating Investigator (PCI):

Prof. Dr. Antonia M. Joussen

Charité

Department of Ophthalmology

Hindenburgdamm 30

12200 Berlin, Germany

Trial protocol code: RadiRet

DRKS: 00003246

EudraCT number: 2011-004463-69

Version of 04 December~~18 July~~ 2012, Version v4-02-F~~3-10-F~~

The information in this trial protocol is strictly confidential. It is for the use of the sponsor, investigator, trial personnel, ethics committee, the authorities, and trial subjects only. This trial protocol may not be passed on to third parties without the express agreement of the sponsor or the Principal Coordinating Investigator (PCI, "Leiter der klinischen Prüfung (LKP)").

This protocol was written based on the template provided by G. Grass and C. Weiß.

Signatures

Prof. Dr. med. Antonia M. Joussen

(Principal Coordinating Investigator, PCI)	Signature	Date

Charité, Department of Ophthalmology, Berlin; on behalf of the sponsor

Prof. Dr. med. Norbert Bornfeld

(Co-Principal Investigator, CPI)	Signature	Date

University of Essen, Department of Ophthalmology, Essen

PD Dr. Martin Hellmich

(Statistician)	Signature	Date

University of Cologne, Institute of Medical Statistics, Informatics and Epidemiology, Cologne

I. Synopsis

Sponsor:	Charité, University Medicine Berlin<br>Schumannstr. 20/2110117 Berlin, Germany<br><br>Represented by:<br>Prof. Dr. med. Antonia M. Joussen (Principal Coordinating Investigator, PCI)<br>Charité University Medicine Berlin<br>Department of Ophthalmology<br>Hindenburgdamm 30<br>12200 Berlin, Germany
Principal Coordinating Investigator:	Prof. Dr. med. Antonia M. Joussen
Title of the clinical trial:	Investigation of Radiation Retinopathy (RadiRet Study)<br>Subtitle: Influence of Lucentis® on radiation retinopathy after irradiation of choroidal melanoma
Indication:	Radiation Retinopathy
Phase:	Phase II, therapeutic-exploratory
Type of trial, trial design, methodology:	Bi-centre, two-arm, randomised, parallel-group, observer-masked, controlled clinical trial in Germany under the auspices of retina.net
Number of subjects:	30 patients per treatment arm (total 60)

Primary trial objective:	To demonstrate superiority of ranibizumab 0.5 mg to laser treatment regarding change from baseline in BCVA over a 6-month treatment period
Study endpoints:	Primary endpoint:<br>• Change from baseline in BCVA over 6 months (area under curve / 6 months)<br>Secondary endpoints:<br>• Change from baseline in macular thickness and the size of areas of macular and peripheral capillary drop out over 6 months (area under the curve / 6 months)<br>• Change from baseline in BCVA, macular thickness and the size of areas of macular and peripheral capillary drop out over 12 months (area under the curve / 12 months)<br>• Proportion of patients with improvement of visual acuity after 6 and 12 months<br>• Rate of vitreous hemorrhages

Criteria for evaluation:

Efficacy:

- Best corrected visual acuity (BCVA, ETDRS)
- Refraction
- Slitlamp
- Fluorescein angiography
- Fundus photography
- OCT
- NEI-VQF

Safety:

- Tonometry
- See “Efficacy”

Medical condition and Principal inclusion criteria:	Medical condition or disease to be investigated:<br>• Patients with retinopathy, due to irradiation in uveal melanoma<br>Principal inclusion criteria:<br>• Clinical signs of radiation retinopathy (cotton wool spots, hemorrhages, vascular ischemia)<br>• Visual impairment due to focal or diffuse radiation induced macula edema (ME) in the irradiated eye that is eligible for laser treatment<br>• Age ≥18 years<br>• BCVA less than 20/32<br><br>Principal exclusion criteria:<br>• Concomitant conditions in the study eye which could, in the opinion of the investigator, prevent VA improvement, e.g. tumor recurrence, tumor growth underneath the macula<br>• Endoresection and / or previous vitrectomy<br>• Patients with proliferative retinopathies or macular edema due to reasons other than irradiation: e.g. diabetic retinopathy, vein occlusion, or Irvine-Gass syndrome<br>• Treatment with anti-angiogenic drugs or intravitreal corticosteroids or any other investigational drug within 3 months prior to randomisation<br>• Prior laser photocoagulation treatment within 3 months (focal / grid laser) or 6 months (panretinal) prior to study entry<br>• Known hypersensitivity against local anaesthetics<br>• Known hypersensitivity against iodine

Name of investigational medicinal product (IMP):	Ranibizumab (Lucentis®) intravitreal injections
Investigational medicinal product – dosage and method of administration:	Ranibizumab - three initial monthly Lucentis® intravitreal injections (0.5 mg). Subsequent injections if visual acuity drops by > 5 letters from best observed on treatment (including baseline) value and evidence of macular or optic disc edema
IMP or therapy used as a comparator – dosage and method of administration:	Laser treatment of the macula and periphery; additional treatment at intervals of not less than 3 months if visual acuity drops by > 5 letters from best observed on treatment (including baseline) value and evidence of ischemic areas, macular or optic disc edema is present
Duration of treatment:	6 months treatment period with either ranibizumab or laser treatment. Total follow-up is 12 months

Time plan:

First patient first visit (FPFV):	1 ~~December~~ April 2013~~2~~
Last patient first visit (LPFV):	1 April~~December~~ 2015~~4~~
Last patient last visit (LPLV):	1 April~~December~~ 2016~~5~~
Final study report:	1 April~~December~~ 2017~~16~~

Statistician:

PD Dr. rer. medic. Martin Hellmich
Institute for Medical Statistics, Informatics and Epidemiology
University of Cologne
Kerpener Str. 62
50937 Cologne
Germany

Statistical methods:	Patients are dynamically assigned to treatment arms using Pocock's minimisation method with random element (radiation dose to the macula and disc, tumor location (anterior/equator/posterior) and study centre (Berlin/Essen) as factors) as implemented by a 24/7 Internet service.<br><br>Outcome assessors are masked to the treatment assignment.<br><br>The primary analysis is according to intention-to-treat (no exclusions). An analysis of covariance with baseline and centre as covariate (type II SS) is applied to the primary variable/endpoint. Missing values are imputed by the last observed value (possibly baseline). Robustness of results to various imputation approaches (best case, worst case, multiple imputation and pooling) are investigated in sensitivity analyses. MMRM analyses are to complement the findings.<br><br>Analyses of secondary variables / endpoints follow the same lines. Safety data are summarized (multple-way contingency tables) and listed.
GCP conformance:	The present trial will be conducted in accordance with the valid versions of the trial protocol and the internationally recognised Good Clinical Practice Guidelines (ICH-GCP), including archiving of essential documents.
Financing:	This investigator initiated trial is financially supported by Novartis Pharma, Nürnberg, Germany. Trial Sponsorship is taken over by Charité University Medicine Berlin.

II. Table of contents

I. Synopsis 3

II. Table of contents 9

II.a) List of tables 13

II.b) List of figures 14

III. Abbreviations 15

1. Introduction 16

2. Objectives of the clinical trial 18

2.1. Rationale for the clinical trial 18

2.2. Primary objective 23

2.3. Secondary and other objectives 24

3. Organisational and administrative aspects of the trial 25

3.1. Sponsor 25

3.2. Principal Coordinating Investigator 25

3.3. Statistics 26

3.4. Data Monitoring Committee 26

3.5. Further committees 26

3.5.1. Steering Committee 26

3.5.2. Advisory Committee 26

3.5.3. Review Board 27

3.6. Study laboratories and other technical services 27

3.7. Central organisation units 27

3.8. Investigators and trial sites 28

3.9. Financing 29

4. Trial conduct 30
4.1. General aspects of trial design 30
4.1.1. Time plan 30
4.2. Discussion of trial design 31
4.3. Selection of trial population 32
4.3.1. Inclusion criteria 32
4.3.2. Exclusion criteria 32
4.4. Withdrawal of trial subjects after trial start 34
4.4.1. Procedures for premature withdrawal from treatment during the trial 34
4.5. Closure of trial sites/Premature termination of the clinical trial 35
4.5.1. Closure of trial sites 35
4.5.2. Premature termination of trial 35
4.6. Treatment 36
4.6.1. Treatments to be given 36
4.6.2. Description of investigational medicinal product 38
Lucentis® 38
4.6.2.1. Manufacture of the investigational medicinal product 38
4.6.2.2. Labelling of investigational medicinal product 38
4.6.2.3. Storage of investigational medicinal product 38
4.6.3. Compliance with treatment / Dispensing and return of investigational medicinal product 39
4.6.4. Assignment of trial subjects to treatment groups 39
4.6.5. Selection of dosage of investigational medicinal product 39
4.6.6. Time of administration and adjustments to dosage of the investigational medicinal product in the individual trial subject 39

4.6.7. Blinding 39

4.6.8. Previous and concomitant medication 40

4.6.8.1. Rescue therapy for emergencies 40

4.6.9. Continuation of treatment after the end of the clinical trial 40

4.7. Efficacy and safety variables 40

4.7.1. Measurement of efficacy and safety variables 40

4.7.1.1. Primary target variable 40

4.7.1.2. Secondary and other target variables 41

4.7.1.3. Safety analysis 42

4.7.1.4. Description of visits 44

4.7.2. Rationale for assessment procedures 47

4.7.3. Pharmacokinetics/Determination of drug levels 47

4.8. Data quality assurance 48

4.8.1. Monitoring 48

4.8.2. Audits/Inspections 49

4.9. Documentation 49

4.9.1. Data management 49

4.9.2. Archiving 50

5. Ethical and regulatory aspects 51

5.1. Independent ethics committee 51

5.2. Ethical basis for the clinical trial 51

5.2.1. Legislation and guidelines used for preparation 51

5.3. Notification of the authorities, approval and registration 52

5.4. Obtaining informed consent from trial subjects 52

5.5. Insurance of trial subjects 53

5.6. Data protection 53

6. Statistical methods and sample size calculation 54

6.1. Statistical and analytical plan 54

6.1.1. Trial populations 54

6.1.2. Description of trial subject groups 54

6.1.3. Primary target variable 55

6.1.4. Secondary target variables 55

6.1.5. Subgroup analyses 55

6.1.6. Interim analysis 55

6.2. Sample size calculation 55

7. Safety 57

7.1. Definitions of adverse events and adverse drug reactions 57

7.1.1. Adverse event 57

7.1.2. Adverse drug reaction 58

7.1.3. Serious adverse events and serious adverse reactions 58

7.1.4. Unexpected adverse drug reaction 59

7.1.5. Suspected unexpected serious adverse reactions 59

7.1.6. Other possible trial-specific complications or risks 59

7.2. Documentation and follow-up of adverse events 61

7.2.1. Documentation of adverse events and adverse drug reactions 61

7.2.2. Severity of the adverse event 62

7.2.3. Causal relationship between adverse event and investigational medicinal product 63

7.3. Reporting of serious adverse events, pregnancy and changes in risk-benefit assessment 64

7.3.1. Reports from the investigator to the sponsor 65

7.3.2. Unblinding when treatment is blinded 65

7.3.3. Notification of ethics committee and competent supreme federal authority 66

7.3.4. Review and reporting of changes in the risk-benefit ratio 66

7.3.5. Informing the Data Monitoring Committee 67

7.3.6. Informing the investigators 67

7.3.7. Informing the marketing authorisation holder 67

7.4. Annual safety report of trial subjects 68

8. Use of trial findings and publication 69

8.1. Reports 69

8.1.1. Interim reports 69

8.1.2. Final report 69

8.2. Publication 69

9. Amendments to the trial protocol 71

10. References 72

11. Appendices 78

11.1. Trial sites and principle investigators 78

II.a) List of tables

Table 1: Time plan of the trial 30

Table 2: Investigations during the clinical trial 44

Table 3: Visit schedule 46

II.b) List of figures

Figure 1: RESTORE-Study (Mitchell 2011) 22

Figure 12: Trial flowchart 31

III. Abbreviations

abbreviation	meaning
AE	Adverse Event
BfArM	Federal Institute for Drugs and Medical Devices (Bundesinstitut für Arzneimittel und Medizinprodukte)
CRF	Case Report Form
DMC	Data Monitoring Committee
DME	Diffuse macula edema
LKP	Principal Coordinating Investigator (Leiter der klinischen Prüfung)
LOCF	Last observation carried forward
ME	Macula edema
PEI	Paul-Ehrlich-Institut
PRN	pro re nata (= when necessary)
PRP	pan retinal photocoagulation
SAE	Serious Adverse Event
SUSAR	Suspected Unexpected Serious Adverse Reaction

1. Introduction

Uveal melanoma (UM) is the most common primary intraocular tumour in adults and occurs in 7-10 cases per million people per year in Caucasian populations (Wilkes 1979).

In case of a primary UM, different treatments are available. Tumors with a thickness < 8 mm can be treated with proton beam therapy, stereotactic irradiation, or by a combination of radiation therapy and local resection or a combination with transpupillary thermotherapy (TTT).

Several complications are related to radiation therapy, including radiation retinopathy. Radiation retinopathy is a *delayed-onset disease* of retinal blood vessels due to alterations in the vessel structure and their permeability after radiation. Retinal ischemia and leaking vessels are the two most significant clinical characteristics of radiation retinopathy. Unfortunately, treatment options that will stop or reverse vision loss due to radiation retinopathy are very limited. There is no “standard of care” (Wen et 2009), however, laser photocoagulation is the most frequent therapy for the peripheral ischemic areas, with a macular grid technique for clinically significant radiation retinopathy.

VEGF (Vascular Endothelial Growth Factor), an angiogenic stimulating factor plays an important role in neovascularization of the retina and vascular leakage leading to macular edema. Drugs that inhibit VEGF are therefore being investigated for their therapeutic potential.

Ranibizumab (Lucentis®) is a recombinant humanized immunoglobulin monoclonal antibody fragment that binds to human VEGF and prevents interaction with its receptors, thereby reducing endothelial cell proliferation, vascular leakage and the formation of new blood vessels (Chen 1999, Gaudreault 2005).

Little information is available regarding the treatment of radiation retinopathy with VEGF inhibitors (Finger et al. 2010). However, in patients with *age-related macular degeneration*, Chang (2007) showed that 95% of those treated with 0.5 mg ranibizumab maintained their visual acuity after 12 months compared to 62% of those receiving sham injections. Furthermore, 34% of patients in the ranibizumab group experienced a clinically significant improvement in vision, compared with 5% in the sham group. By administrating either

Lucentis® or laser coagulation in a randomised study, we hope to determine whether such treatment can diminish or delay the development of complications following radiation therapy, including macular edema and vitreous hemorrhage, thus leading to less loss or even an improvement in visual acuity.

2. Objectives of the clinical trial

2.1. Rationale for the clinical trial

Uveal melanoma (UM) is the most common primary intraocular tumour in adults and occurs in 7-10 cases per million people per year in Caucasian populations (Wilkes 1979).

Most melanocytic tumours of the uveal tract arise from the choroid, which is one of the most capillary-rich tissues of the body. Among the known risk factors related to survival are *tumour cell type, tumour diameter, tumour location, age, and certain chromosomal abnormalities*.

In cases of primary UM, there is a variety of available treatments. For tumors with a thickness of > 8 mm, enucleation is often the first choice, proton beam therapy could be an alternative. Small to medium-sized tumors with a prominence < 8 mm can be treated with proton beam therapy, stereotactic irradiation, or by sandwich therapy, a combination of radiation and laser therapy, using a plaque containing radioactive material which is placed on the sklera overlying the tumor. Proton beam irradiation is the only method that allows the accurate sparing of structures close to the tumor such as the optical nerve and macula.

Radiation therapy results in destruction of the intraocular tumor, either through the direct damage of tumor cell DNA, thus preventing further cell division and growth (Goodman 1986, Char 1982, 1983, 1989), or through damage to capillary endothelium, leading to ischemia and tumor necrosis (Gragoudas 1980, Goodman 1986, Char 1989). Proton therapy is a highly effective local therapy, leading to a local tumor control in more than 95% of patients and a rate of enucleation lower than 14% (Höcht 2004). However, this high success rate has brought the long term complications of radiation therapy in focus often involving a significant deterioration in vision.

These sequelae include tumor hemorrhage, persistent vitreous hemorrhage, cataract, radiation induced optic neuropathy and radiation retinopathy.

Radiation retinopathy is a slowly progressive, delayed-onset disease of retinal blood vessels due to changes in the structure and permeability of the retinal vessels. Ophthalmoscopic and fluorescein-angiographic findings characteristic of radiation retinopathy include macular

changes such as macular edema, capillary non-perfusion, cotton wool spots, capillary telangiectasia, retinal neovascularization, micro- aneurysms, retinal hemorrhages, intraretinal exudation, and neuronal changes such as disc edema, disc pallor, optic nerve atrophy, and neovascularization of the disk (Guyer 1992).

Clinically, these signs are often identical to the findings seen in diabetic retinopathy. Patients with radiation retinopathy can suffer a loss of vision through any of these complications. Currently, there is no treatment to reverse visual loss from ischemic maculopathy due to capillary nonperfusion (Guyer 1992).

The threshold for radiation retinopathy depends on the total dose delivered, the volume of irradiated retina, the fractionation scheme, and individual factors. In general, a greater total dose results in earlier, more severe and more pronounced radiation retinopathy (Harris 1976). *Doses of more than 45 Gy imply an increased risk, and must be expected with doses over 65 Gy* (Parsons 1994).

Proton beam and plaque radiation therapy are now well established as alternatives to enucleation.

Plaque radiotherapy represents a form of brachytherapy, and generally uses iodine-125 and ruthenium-106. Dosimetry during treatment is indirect, as exemplified by the dose calculation formalism used in the protocol of Task Group 43 (TG-43) of the American Association of Physicists in Medicine (AAPM). Because of the relative steep depth dose curves involved in brachytherapy with ruthenium plaques and the requirement of a minimum dose to the tumor apex of 80 – 100 Gy, typical doses to the sclera are high. For example, with iodine-125, γ-emitter, a scleral dose of 400 - 700 Gy is typical for a tumor prominence of 5 - 10 mm. For ruthenium-106, a shorter range β-emitter, the scleral dose is typically 700 - 1000 Gy for a tumor prominence of 2 - 5 mm.

Proton therapy is now performed in more than 20 centres worldwide. In Harvard, for instance, it has been used to treat more than 5000 patients since 1975, in Berlin more than 2000 patients have been treated since 1998. It allows a very precise calculation of the dose to the macula and optic disc as well as the peripheral retina, at the same time as minimising the irradiated volume of the posterior uveal structures. Dosimetry is performed with ionization chambers along the guidelines of Technical Report Series No. 398 by the International

Atomic Energy Agency (IAEA). Furthermore, the maximal dose to the retina is 60 CGE (1 CGE = 1 Cobalt Gray Equivalent - dose which effect is equivalent to 1 Gy of Cobalt 60 radiation) , far lower than that arising from plaques. Proton therapy thus carries an inherently lower risk of retinal radiation retinopathy of especial importance with regard to tumors proximal to central structures (Höcht et. al.2004).

To date, there is no effective therapy to cure vision loss due to radiation retinopathy. Cystoid macular edema may benefit from a macular grid treatment. If there are localized capillary changes causing exudative retinopathy, the focal laser treatment of these areas may decrease macular edema. In many patients, maculopathy after radiation progresses from a leaking exudative phase to an occlusive phase, and focal grid photocoagulation might serve only as a temporary measure. Panretinal photocoagulation appears to be beneficial in reducing neovascularization of the disc and retina as well as rubeosis iridis (Gass 1968, Chaudhuri 1981, Finger 2005).

Retinal ischemia and leaking vessels are the two most significant factors in radiation retinopathy. Neovascularization, like leaking vessels, depends on the balance between angiogenic stimulators and inhibitors. Microangiopathy impairs vascularization of the retina, which causes subsequent permanent damage neuroretinal structures due to ischemia. As a consequence, the production of angiogenic stimulating factors will increase (Ferrara 1997, Neufeld 1999, Witmer 2003). The most essential angiogenic stimulating factors are VEGF and bFGF. Increased concentrations of VEGF-A have been detected in the anterior chamber of the eye in patients with uveal melanoma (Missotten 2006), and extraordinarily high concentrations were found in eyes that underwent enucleation for complications after irradiation. VEGF plays an important role in pathological angiogenesis, and increases retinal capillary permeability by breaking down the blood–retina barrier. Higher concentrations of VEGF were also found in the aqueous humor of patients with diabetic retinopathy and macular edema versus healthy eyes (Funatsu 2005).

Ranibizumab (Lucentis) is a monoclonal antibody (Fc-fragment) that inhibits the action of VEGF-A. It binds to human VEGF and prevents interaction with its receptors VEGFR1 and VEGFR2 on the surface of endothelial cells, thereby *reducing endothelial cell proliferation, vascular leakage and the formation of new blood vessels* (Chen 1999, Gaudreault 2005). Finger reported on a single case of radiation optic neuropathy that improved after an

intravitreal injection of bevacizumab (Avastin), and on 8 cases with radiation macular edema, some of which improved after bevacizumab (Finger 2007).

Ranibizumab is a recombinant humanized immunoglobulin monoclonal antibody fragment designed for intraocular use. The efficacy and safety of ranibizumab were evaluated in several randomised trials involving 1,300 patients with neovascular age-related macular degeneration. In the first trial, patients with minimally classic or occult choroidal neovascular (CNV) lesions received monthly ranibizumab 0.3 mg or 0.5 mg intravitreal injections or monthly sham injections for 24 months. Chang (2007) demonstrated that in 95% of patients treated with 0.5 mg ranibizumab, visual acuity after 12 months remained stable, compared with 62% of those receiving sham injections. As ranibizumab has been shown to be very effective in the treatment of macular degeneration, we wonder whether its use will help to improve vision in cases of radiation retinopathy. First results of clinical trials demonstrate promising results using VEGF inhibitors: bevacicumab has beein used to treat radiation neuropathy and retinopathy (Finger 2007 a, b, 2008, Gupta 2008, Mason 2007, Wen 2009, Ziemssen 2007). Similarly, first results using ranibizumab have been reported (Dunavoelgyi 2007, Finger 2010).

Diabetic macular edema and diabetic retinopathy demonstrates similar features as radiation retinopathy. For diabetic macular edema several studies have shown the benefit of anti-VEGF treatment and ranibizumab is approved for clinical treatment. Thus, the likelihood is high that ranibizumab is similarly efficient for radiation retinopathy and macular edema.

The RESTORE-Study (Mitchell 2011) demonstrated the benificial effect of repetetive injections of ransibizumab on diabetic macular edema (phase III clinical trial).

There is an increase in visual acuity over the first year that is maintained over 3 years. The group receiving laser treatment initially received randibizumag in the second and third year of the study with a considerable improvement in visual acuity:

Figure 1: RESTORE-Study (Mitchell 2011)

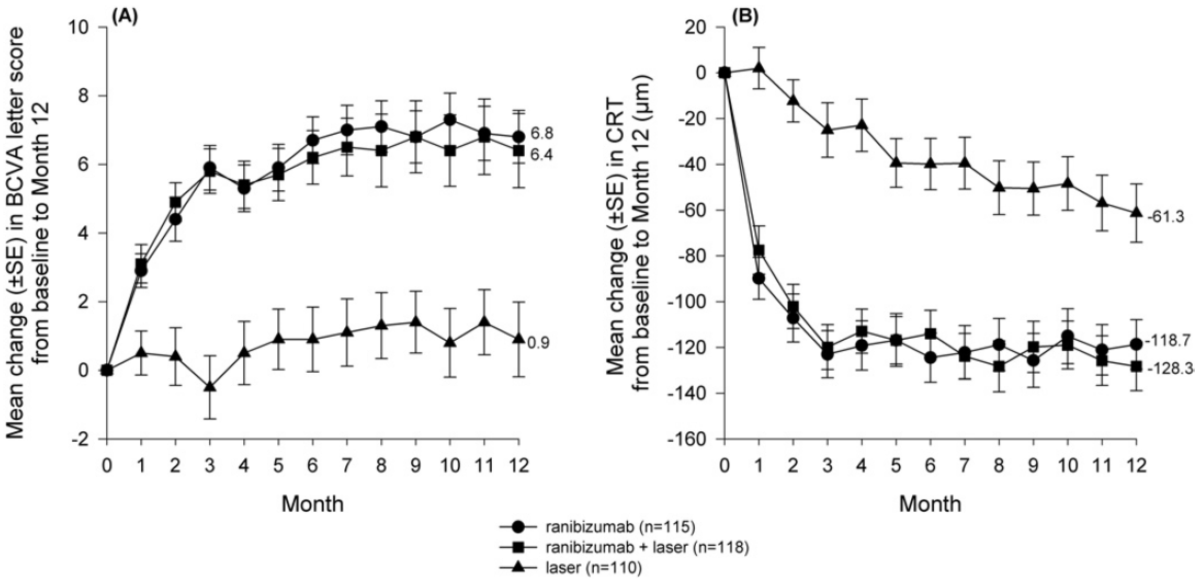

Similar effects of ranibizumab are exepected for radiation retinopathy. As the disease is usually self-limiting after 2-3 years, we expect that the total number of injections needed is much fewer compared to diabetic retinopathy.

In conclusion, the use of anti-VEGF for active radiation retinopathy is advantageous over pure observation or photocoagulation as it may allow for vascular remodelling and faster reduction of the macular edema.

Implications

Approximately 30-40% of patients develop a deterioration of visual acuity *within 5 years* after treatment of uveal melanoma using radiation therapy due to radiation retinopathy (Shields 2002, Bartlema 2003). By administration of either Lucentis® we hope to treat complications of radiation therapy, by demonstrating a lack of further decrease in best-corrected visual acuity and a reduced amount of macular edema and vascular leakage. Additionally, we hope to obtain a better understanding of the pathophysiologic processes involved, by demonstrating a *possible relation between high levels of angiogenic factors (VEGF) in the anterior chamber fluid, and radiation retinopathy*. In conclusion, we hope to provide evidence for a new therapy in patients with retinopathy, due to radiation in uveal melanoma. There is no scientifically proven treatment available at this time although laser photocoagulation is considered the standard of care.

2.2. Primary objective

Approximately 30-40% of patients develop a deterioration of visual acuity within 5 years after the radiotherapy of uveal melanoma due to radiation retinopathy (Shields 2002, Bartlema 2003).

By administration of Lucentis® (ranibizumab) we hope to demonstrate a relevantly improved visual acuity and a reduced amount of macular edema and vascular leakage within a period of ~~12~~ 6 months as compared to laser coaguagulation alone. Additionally, we hope to obtain a better understanding of the pathophysiologic processes involved, by demonstrating a possible relation between high levels of angiogenic factors (VEGF) in the anterior chamber fluid and radiation retinopathy. *In conclusion, we hope to provide evidence for a new and effective treatment for uveal melanoma patients with retinopathy following radiotherapy, where no scientifically proven treatment yet exists.*

1. VEGF plays an important role in pathological angiogenesis and vessel leakage. The hypothesis is that application of a VEGF inhibitor, specifically Lucentis®, will have a

beneficial effect on reducing the neovascularization and leakage of vessels in radiation retinopathy, *similar* to its proven effect in age-related macular degeneration.

2. Photocoagulation is the standard of care in radiation retinopathy. The effect on maular edema is limited and needs to be analyzed against treatment with Lucentis®.

The study **aims** are therefore:

1. To demonstrate a positive effect of a new treatment for patients with retinopathy, due to radiation in uveal melanoma on formation of the avascular zone and macular edema. There is no scientifically proven treatment available at this time.
2. To obtain a preservation and/or improvement of visual acuity in patients with radiation retinopathy after treatment.

Primary objective / Hypothesis:

To demonstrate superiority of ranibizumab 0.5 mg to laser treatment regarding change from baseline in BCVA over a 6-month treatment period.

2.3. Secondary and other objectives

The secondary objectives of the trial are:

To evaluate whether ranibizumab 0.5 mg as monotherapy is superior to laser treatment with regard to

- Change from baseline in macular thickness and the size of areas of macular and peripheral capillary drop out over 6 months (area under the curve / 6 months)
- Change from baseline in BCVA, macular thickness and the size of areas of macular and peripheral capillary drop out over 12 months (area under the curve / 12 months)
- Proportion of patients with improvement of visual acuity after 6 and 12 months
- Rate of vitreous hemorrhages

3. Organisational and administrative aspects of the trial

3.1. Sponsor

Sponsor : Charité, University Medicine Berlin
Hindenburgdamm 30
12200 Berlin, Germany

Represented by: Prof. Dr. med. Antonia M. Joussen
(Principal Coordinating Investigator [PCI])
Charité University Medicine Berlin
Department of Ophthalmology
Hindenburgdamm 30
12200 Berlin, Germany

3.2. Principal Coordinating Investigator

Principal Coordinating
Investigator (PCI): Prof. Dr. med. Antonia M. Joussen
Charité University Medicine Berlin
Department of Ophthalmology
Hindenburgdamm 30
12200 Berlin, Germany
E-Mail: joussena@googlemail.com
Phone: +49 30 8445 2331

Co-Principal Coordinating
Investigator (PCI): Prof. Dr. med. Norbert Bornfeld
University of Essen

Department of Ophthalmology
Hufelandstr. 55
45122 Essen, Germany
nbornfeld@googlemail.com

3.3. Statistics

Statistician: PD Dr. rer. medic. Martin Hellmich
Institute for Medical Statistics, Informatics and Epidemiology
University of Cologne
Kerpener Str. 62
50937 Cologne
Germany

3.4. Data Monitoring Committee

There will be no Data Monitoring Committee set up in this clinical trial.

3.5. Further committees

3.5.1. Steering Committee

There will be no Steering Committee set up in this clinical trial.

3.5.2. Advisory Committee

There will be no Advisory Committee set up in this clinical trial.

3.5.3. Review Board

Best corrected visual acuity (BCVA), size of areas of macular and peripheral capillary drop-out, macula thickness will be analyzed in a blinded fashion by Dr. Florian Heussen and Dr. Yanling Ouyang, Charité.

3.6. Study laboratories and other technical services

There are no other tasks that will be performed by other service providers.

3.7. Central organisation units

Project management: Cologne Centre for Clinical Trials (ZKS Köln)
Gleueler Strasse 269
50935 Cologne
Germany
Tel.: +49 221 478 88121
Fax: +49 221 478 7983

Monitoring: Cologne Centre for Clinical Trials (ZKS Köln)
Gleueler Strasse 269
50935 Cologne
Germany
Tel.: +49 221 478 88121
Fax: +49 221 478 7983

Data management: Cologne Centre for Clinical Trials (ZKS Köln)
Gleueler Strasse 269
50935 Cologne
Germany
Tel.: +49 221 478 88121
Fax: +49 221 478 7983

SAE management: Cologne Centre for Clinical Trials (ZKS Köln)
Gleueler Strasse 269
50935 Cologne
Germany
Tel.: +49 221 478 88121
Fax: +49 221 478 7984

Scientific advice: Cologne Centre for Clinical Trials (ZKS Köln)
Gleueler Strasse 269
50935 Cologne
Germany
Tel.: +49 221 478 88121
Fax: +49 221 478 7983

3.8. Investigators and trial sites

This clinical trial will be carried out as observer-masked trial at two trial sites in Germany. If necessary, further qualified trial sites may be recruited to the trial.

A list of trial sites involved, including information on the principal investigators, further investigators, and trial staff, will be kept and continuously updated. A list of the trial sites with names of the principal investigators is given in Appendix 11.1.

Requirements for investigators and trial sites

The investigators should be familiar with intravitreal injection procedures as well as macular laser. The invesigators and trial sites have to proof knowledge of regulatory procedures, and

experience with the conduct of the clinical testing of pharmaceutical preparations. They are required to have special experience in the indication of radiation retinopathy. The principal invectigator herself has to be an investigator and must have at least 2 years of experience in the clinical testing of pharmaceutical preparations.

3.9. Financing

This investigator initiated trial is financially supported by Novartis Pharma, Nürnberg, Germany. Trial Sponsorship is taken over by Charité University Medicine Berlin.

4. Trial conduct

4.1. General aspects of trial design

This is a bi-centre, phase II, two-arm, randomised, parallel-group, observer-masked, controlled clinical trial.

4.1.1. Time plan

Table 1: Time plan of the trial

First patient first visit (FPFV):	1 December April 20132
Last patient first visit (LPFV):	1 AprilDecember 20154
Last patient last visit (LPLV):	1 AprilDecember 20165
Final study report:	1 AprilDecember 20176

End of the clinical trial

The end of the trial is defined as the last visit of the last patient included (LPLV).

Figure ~~1~~2: Trial flowchart

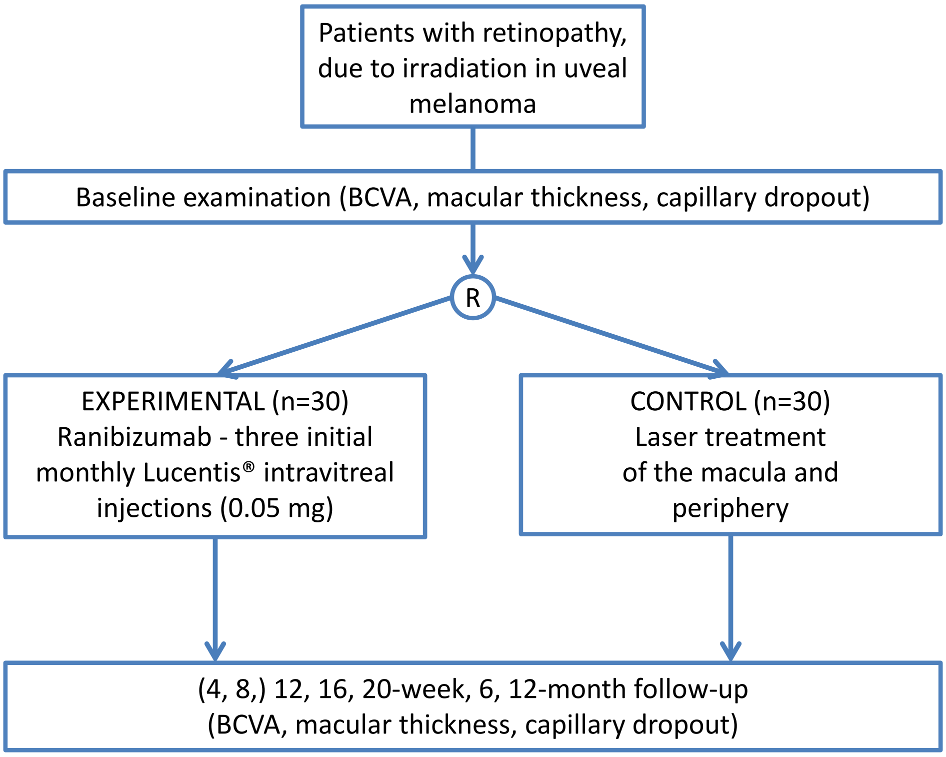

4.2. Discussion of trial design

Selection bias is minimised by concealed random assignment of trial treatments. Balance of parallel treatment groups regarding important prognostic characteristics is assured by Pocock's minimisation procedure with random element (dynamic allocation). Observer-masking (assessment of BCVA, macular thickness, capillary drop-out) is done to guard against *detection bias*. Though sham procedures for intraocular injection and retinal laser treatment are possible (in order to mask patients), these will/can not be implemented in this phase II trial. Generally patients feel the difference between sham injections and true injections as well as between sham laser and true laser treatment due to pain perception. Thus, patients and treating physicians are very likely to guess what has actually been done. *Performance bias* is thus to be reduced by strict standardisation of trial procedures. *Attrition bias* will be minimised by active follow-up of trial patients by a dedicated study nurse / physician (telephone, mail and in-person contact).

4.3. Selection of trial population

Reasons for gender distribution

We expect a homogenous (1:1) gender distribution. There is no evidence of difference by gender in the incidence of choroidal melanoma, nor in the development of radiation retinopathy, nor in the response to anti-VEGF agents or laser treatment.

4.3.1. Inclusion criteria

- Patients with retinopathy, due to radiation of uveal melanoma
- Clinical signs of radiation retinopathy (cotton wool spots, hemorrhages, vascular ischemia
- Visual impairment due to focal or diffuse ME in the irradiated eye that is eligible for laser treatment
- Age ≥18 years
- BCVA less than 20/32
- Not legally incapacitated
- Written informed consent

4.3.2. Exclusion criteria

- Participation in other interventional trials
- Concomitant conditions in the study eye which could, in the opinion of the investigator, prevent VA improvement, e.g. tumor recurrence, tumor growth underneath the macula
- Endoresection and / or previous vitrectomy
- Patients with proliferative retinopathies or macular edema due to reasons other than irradiation: e.g. diabetic retinopathy, vein occlusion, or Irvine-Gass syndrome
- Treatment with anti-angiogenic drugs or intravitreal corticosteroids or any other investigational drug within 3 months prior to randomisation

- Prior laser photocoagulation treatment within 3 months (focal / grid laser) or 6 months (panretinal) prior to study entry
- Known hypersensitivity against local anaesthetics
- Known hypersensitivity against iodine
- Contraindications in Information Sheet for Health Professionals (Summary of medicinal Product Characteristics, SmPC; Fachinformation in Germany) or Investigator’s Brochure
- Pharmaceutical preparations with which interactions can be expected
- Diseases or findings that may have a significant effect on the target variables and which may therefore mask or inhibit the therapeutic effect under investigation
- Pregnant or nursing women
- Failure to use highly-effective contraceptive methods. The following contraceptive methods with a Pearl Index lower than 1% are regarded as highly-effective:
    - Oral hormonal contraception (‘pill’)
    - Dermal hormonal contraception
    - Vaginal hormonal contraception (NuvaRing®)
    - Contraceptive plaster
    - Long-acting injectable contraceptives
    - Implants that release progesterone (Implanon®)
    - Tubal ligation (female sterilisation)
    - Intrauterine devices that release hormones (hormone spiral)
    - Double barrier methods

  This means that the following are not regarded as safe: condom plus spermicide, simple barrier methods (vaginal pessaries, condom, female condoms), copper spirals, the rhythm method, basal temperature method, and the withdrawal method (coitus interruptus).

- Persons with any kind of dependency on the investigator or employed by the sponsor or investigator
- Persons held in an institution by legal or official order

4.4. Withdrawal of trial subjects after trial start

A study subject will be early discontinued from participation in the study if

- They withdraw consent from participating in the study
- Any clinical adverse event (AE), laboratory abnormality, illnes or other medical condition or situation occurs that would not go along with the best interest of the patient in case of continuing the participation. This may be tumor recurrence, drop of visual acuity below CF, or requirement for ocular surgical treatment, e.g. in case of retinal detachment, endophthalmitis or other serious ocular adverse events. Similarly systemic conditions leading to withdrawal of the trial subject are possible and may comprise e.g. systemic metastesis or other severe systemic conditions affecting the patient general health in a way that prevents him or her to participate in the study. Patients presenting with newly diganosed symptoms of vascular disease such as coronary heart diease or stroke that occurs during the treatment will be considered severe adverse events and excluded from the study.
- Systemic metastesis of choroidal melanoma results in immeditate drop out of the patient from the trial. Systemic metastesis of choroidal melanoma is up to now not sufficiently treatable and has a limited prognosis in patient survival. Note: Since the primary analysis is according to the intention-to-treat, each patient should be followed-up even if the study treatment is discontinued.

4.4.1. Procedures for premature withdrawal from treatment during the trial

In case of patient withdrawal during the study period all data collected until this point of time will be stored according to AMG §40, 2a, 3. The patient must not be replaced.

4.5. Closure of trial sites/Premature termination of the clinical trial

4.5.1. Closure of trial sites

A trial site will be excluded in case of insufficient or lack of recruitment or insufficient documentation, or decision of local site investigator, Steering Committee, Principal Coordinating Investigator [PCI] or sponsor.

4.5.2. Premature termination of trial

The sponsor has the right to terminate the trial prematurely if there are any relevant medical or ethical concerns, or if completing the trial is no longer practicable. If such action is taken, the reasons for terminating the trial must be documented in detail. All trial subjects still under treatment at the time of termination must undergo a final examination which must be documented. The PCI must be informed without delay if any investigator has ethical concerns about continuation of the trial.

Premature termination of the trial will be considered if:

- The risk-benefit balance for the trial subject changes markedly
- It is no longer ethical to continue treatment with the IMP
- The sponsor considers that the trial must be discontinued for safety reasons
- An interim analysis or results of other research show that one of the trial treatments is superior or inferior to another
- It is no longer practicable to complete the trial

The sponsor decides on whether to discontinue the trial in consultation with the PCIand/or statistician.

4.6. Treatment

4.6.1. Treatments to be given

Formulation of Ranibizumab

Ranibizumab is formulated as a sterile solution aseptically filled in a sterile, 3-mL stoppered glass vial. Each vial contains 0.5 mL of 10 mg/mL (0.5-mg dose level) ranibizumab aqueous solution (pH 5.5) with histidine, trehalose, and polysorbate 20. The vial contains no preservative and is suitable for single use only. Vials should be protected from direct sunlight.

Dosage and administration of Ranibizumab

Ranibizumab will be administered intravitreally in a multiple-dose regimen of 0.5 mg of ranibizumab every month (Days 0, 30, 60) for a total of 3 injections and as a PRN Treatment thereafter. Subsequent injections if visual acuity drops by > 5 letters from best observed on treatment (including baseline) value or evidence of macular or optic disc edema.

Injection is discontinued when

- No further BCVA improvement due to treatment at 2 last consecutive visits or
- BCVA≥84 letters at 2 last consecutive visits

Monthly injections are continued when

- Decrease in BCVA due to DME in the opinion of the investigator

If a subject is unable to receive study drug because of medical or personal reasons, treatment should be resumed within 2 weeks of the scheduled treatment. If a subject is unable to receive study drug within 2 weeks of the scheduled treatment, the subject will be allowed to miss the scheduled dose and resume treatment at the next regularly scheduled time. Dosing should not occur more frequently than every 14 days. Missed doses will not be replaced.

Ranibizumab Injection

Procedures will be implemented to minimise the risk of potential adverse events associated with serial intraocular injections (e.g., endophthalmitis). Aseptic technique must be observed by clinic staff involved in the injection tray assembly, anesthetic preparation and administration, and study drug preparation and administration. In addition to the procedures outlined in the protocol, added safety measures in adherence to specific institutional policies associated with intraocular injections should be observed.

Ranibizumab will be administered in the study eye only. Intravitreal injection must be performed by the injecting physician(s) following the slitlamp examination and in accordance to standard treatment precautions and aseptic techniques.

A 30-gauge, ½-inch needle, attached to a low-volume (e.g., tuberculin) syringe containing 50 μL of study drug solution, will be inserted through the pre-anesthetized conjunctiva and sclera, approximately 3.5–4.0 mm posterior to the limbus, avoiding the horizontal meridian and aiming toward the center of the globe. Immediately following the intraocular injection, standard topical treatment for intraocular injections is administered and will be continued by the patient according to standards.

In the **laser group**, laser treatment using an Argon Green Laser will be performed to the macula and periphery in areas of ischemia. In the periphery PRP is performed according to the use of PRP in diabetic patients. In the macular area, a modified grid technique is used sparing the fovea; additional treatment at intervals of not less than 3 months if visual acuity drops by >5 letters from best observed on treatment (including baseline) value and evidence of ischemic areas, macular or optic disc edema is present.

Laser (focal or grid or panretinal photocoagulation):

- If deemed necessary by the evaluating investigator and in accordance with the ETDRS guidelines at intervals of ≥3 months from the last treatment

4.6.2. Description of investigational medicinal product

The investigational drug is ranibizumab (see above). Ranibizumab is provided by Novartis. It is approved for intravitreal injection in wet age related macula degeneration, diabetic retinopathy and retinal vein occlusions.

Lucentis®

Ranibizumab (Lucentis®) binds to the receptor binding site of active forms of VEGF-A, including the biologically active, cleaved form of this molecule, $VEGF_{110}$. VEGF-A has been shown to cause neovascularization and leakage in models of ocular angiogenesis. The binding of ranibizumab to VEGF-A prevents the interaction of VEGF-A with its receptors (VEGFR1 and VEGFR2) on the surface of endothelial cells, reducing endothelial cell proliferation, vascular leakage, and new blood vessel formation. The most common adverse events following intra-ocular injection are conjunctival hyperemia and subconjunctival hemorrhage. Serious adverse events related to the injection procedure have occurred in < 0.1% of intravitreal injections, including endophthalmitis, rhegmatogenous retinal detachments, and iatrogenic traumatic cataracts (Heier 2006).

4.6.2.1. Manufacture of the investigational medicinal product

The drug supplied for this study will be manufactured by Novartis.

4.6.2.2. Labelling of investigational medicinal product

The drug supplied for this study will be labeled by Novartis.

4.6.2.3. Storage of investigational medicinal product

The trial medication will be kept separate from the normal medication distribution. The medication is provided by Novartis Pharma and will be distributed to the respective hospital pharmacies that are responsible for storage of the medication in accordance to the study protocol and applicable laws. The IMP will be stored in a secure, limited-access location according to the summary of product characteristics (SMPC).

4.6.3. Compliance with treatment / Dispensing and return of investigational medicinal product

The local coordinating investigator or his/her designee of each trial site will document inventoried and disposed IMP for each site and subject. Destruction of IMPs will be performed by the trial sites and will be documented. In the event that the IMP is received in a damaged condition (i.e. after shipment) or the expiration date of the IMP has past, the study drug should be held in quarantine and not dispensed to patients. In this case the local pharmacy should be informed by the trial site personal and additional drug supplies will be requested for the trial site.

4.6.4. Assignment of trial subjects to treatment groups

Patients will be dynamically allocated to treatment arms using Pocock's minimisation method with random element (Pocock 1975) as implemented by 24/7 Internet service. Minimisation factors include radiation dose to the macula and disc, tumor location (anterior / equator / posterior) and study centre (Berlin / Essen).

4.6.5. Selection of dosage of investigational medicinal product

See 4.6.1

4.6.6. Time of administration and adjustments to dosage of the investigational medicinal product in the individual trial subject

No dosage adjustments are planned in this clinical trial.

4.6.7. Blinding

Observer-masking (assessment of BCVA, macular thickness, capillary drop-out) is done to guard against detection bias. Though sham procedures for intraocular injection and retinal laser treatment are possible (in order to mask patients), these will/can not be implemented in this phase II due to the high likelihood of the patient knowing by a short local pain sensation during each treatment whether it is a sham treatment or a real therapy.

Unblinding

Not applicable (treatment not blinded)

4.6.8. Previous and concomitant medication

Permitted concomitant therapies include cataract surgery for radiation induced cataracts. Other surgical therapies, e.g. vitrectomy are not permitted during the study and will be considered adverse events and treatment failure.

Similarly, other pharmacological treatments for macular edema, e.g. triamcinolone or other steroids are not permitted during the study period.

4.6.8.1. Rescue therapy for emergencies

There is no current standard treatment for radiation retinopathy. Laser treatment for peripheral and central ischemia in the injection group will be considered rescue treatment.

In cases of severe vitreous hemorrhage, vitrectomy and intraoperative laser treatment will be performed as a rescue treatment.

4.6.9. Continuation of treatment after the end of the clinical trial

It is expected that active radiation retinopathy is transitory disease.

4.7. Efficacy and safety variables

4.7.1. Measurement of efficacy and safety variables

4.7.1.1. Primary target variable

Primary variable

- Change from baseline in BCVA over 6 months (area under curve / 6 months)

4.7.1.2. Secondary and other target variables

Secondary variables

- Change from baseline in macular thickness and the size of areas of macular and peripheral capillary drop out over 6 months (area under the curve / 6 months)
- Change from baseline in BCVA, macular thickness and the size of areas of macular and peripheral capillary drop out over 12 months (area under the curve / 12 months)
- Proportion of patients with improvement of visual acuity after 6 and 12 months
- Rate of vitreous hemorrhages

Fundus photography

At every time point, digital colour fundus photos should be made of the central area and every quadrant; central, nasal, nasal-superior, superior, temporal-superior, temporal, temporal-inferior, inferior, nasal-inferior (a total of nine photos, with an optional overview photo (compilation of the nine photos)).

Visual Function Questionaire (VFQ-25)

"The VFQ-25 is a reliable and valid 25-item version of the 51-item National Eye Institute Visual Function Questionnaire (NEI-VFQ). It is especially useful in settings such as clinical trials, where interview length is a critical consideration." (see http://www.rand.org/health/surveys_tools/vfq.html; scoring instructions are described in the manual)

Optical Coherence Tomography (OCT)

Optical Coherence Tomography, or OCT, is a non-contact, non-invasive imaging technique used to obtain high resolution cross-sectional images of the retina. OCT is analogous to ultrasound B-scan imaging except that light rather than sound waves

are used to obtain a much higher longitudinal resolution of approximately 10µm in the retina. OCT has been shown to be clinically useful for imaging selected macular diseases including macular holes, macular edema, age-related macular degeneration, and central serous chorioretinopathy

An OCT of Carl Zeiss (Stratus, Dublin, CA) should be used to perform the measurements. At 4 weeks, 8 and 12 weeks, and then every three months, OCT macular scans of both eyes (6 scans; horizontal, vertical and a fast macular thickness scan to obtain measurement of macular thickness, at every time point) should be performed.

Measurement of ischemic maculopathy by Fluorescein Angiography
Standardized angiography is performed by fluorescein angiography using a confocal scanning laser ophthalmoscope (Spectralis, Heidelberg Engineering). Besides central images of the macula, the periphery will be covered in an 8 field scheme. The angiograms follow a standardised protocol.
Fluorescein angiograms will include pre-injection reflectance images using the green illumination (514 nm) and autofluorescence images using the blue (488 nm) illumination. Angiographic images will be taken within the first second after dye inflow, and thereafter in 1 second intervals for 15 seconds, 30 seconds, 60 seconds, 120 seconds, 300 seconds, and 600 seconds, after injection. For all central images, the 30° mode will be used. Angiographic images will be recorded with a resolution of 256x256 pixels within the first minute after injection. Thereafter, the 512x512 resolution will be used. Subsequently, peripheral images are taken.

4.7.1.3. Safety analysis

Safety paramters that will be assessed:

- *Intraocular pressure*
- *Endophthalmitis*

- *Rhegmatogenous retinal detachment*
- *Retinal tear*
- *Vitreous hemorrhage*
- *Lens damage*
- *Ocular inflammation*
- *Key arterial thromboembolic events*
- *Death*
- *Nonocular haemorrhage*

4.7.1.4. Description of visits

Table 2: Investigations during the clinical trial

Lucentis Treatment Arm

Visit	1	2	3	4	5	6	7	8
Explanation of Study aspects	X							
Check Inclusion/ Exclusion criteria	X							
Informed consent	X							
Demographic data & Medical history	X							
Concommittant Medication	X	X	X	X	X	X	X	
Randomization	X							
Lucentis Therapy	X	X	X	(X)	(X)	(X)		
AEs/SAEs	X	X	X	X	X	X	X	X
Best corrected visual acuity (ETDRS)	X	X	X	X	X	X	X	X
Refraction	X	X	X	X	X	X	X	X
Tonometry	X	X	X	X	X	X	X	X
Slitlamp	X	X	X	X	X	X	X	X
Fluorescein Angiography	X						X	
Fundus photography	X						X	
OCT	X	X	X	X	X	X	X	X
NEI-VFQ	X						X	X
Study continuation / End of study	X	X	X	X	X	X	X	X

Laser Treatment Arm

Visit	1	2	3	4	5	6
Explanation of Study aspects	X					
Check Inclusion/ Exclusion criteria	X					
Informed consent	X					
Demographic data & Medical history	X					
Concommittant Medication	X	X	X	X	X	
Randomization	X					
Laser Therapy	X	(X)	(X)	(X)		
AEs/SAEs	X	X	X	X	X	X
Best corrected visual acuity (ETDRS)	X	X	X	X	X	X
Refraction	X	X	X	X	X	X
Tonometry	X	X	X	X	X	X
Slitlamp	X	X	X	X	X	X
Fluorescein Angiography	X				X	
Fundus photography	X				X	
OCT	X	X	X	X	X	X
NEI-VQF	X				X	X
Study continuation / End of study	X	X	X	X	X	X

Table 3: Visit schedule

Lucentis Arm

Visit	Trial Day	First day possible	Last day possible	Comments
1	1	1	1	Baseline & 1st Lucentis treatment visit at day 1
2	28±2	26	30	2nd Lucentis treatment visit after 4 weeks
3	56±2	54	58	3rd Lucentis treatment visit after 8 weeks
4	84±2	82	86	1st checkup visit & possible 1st PRN therapy after 12 weeks
5	112±7	105	119	2nd checkup visit & possible 2nd PRN therapy after 16 weeks
6	140±7	133	147	3rd checkup visit & possible 3rd PRN therapy after 20 weeks
7	180±7	173	187	Collection of primary endpoint data after 6 months
8	360±7	353	367	Follow-up visit after 12 months

Laser Arm

Visit	Trial Day	First day possible	Last day possible	Comments
1	1	1	1	Baseline & 1st Laser treatment visit at day 1
2	84±2	82	86	1st checkup visit & possible 1st PRN therapy after 12 weeks
3	112±7	105	119	2nd checkup visit & possible 1st PRN therapy after 16 weeks (if not done at visit 2)
4	140±7	133	147	3rd checkup visit & possible 1st PRN therapy after 20 weeks (if not done at visit 2 or 3)
5	180±7	173	187	Collection of primary endpoint data after 6 months
6	360±7	353	367	Follow Up Visit after 12 months

4.7.2. Rationale for assessment procedures

Macula edema is assessed both anatomically (OCT and FAG) and functionally (visual acuity). Further the ophthalmological assessment includes the tumor control.

From other indications of ranibizumab the duration of the treatment effect was determined as 4 weeks. Thus the intervals for clinical assessment in the current trial were set to 4 weeks.

4.7.3. Pharmacokinetics/Determination of drug levels

Not applicable

4.8. Data quality assurance

4.8.1. Monitoring

The trial sites will be monitored to ensure the quality of the data collected. The objectives of the monitoring procedures are to ensure that the trial subject's safety and rights as a study participant are respected, that accurate, valid and complete data are collected, and that the trial is conducted in accordance with the trial protocol, the principles of GCP and local legislation.

All investigators agree that the monitor regularly visits the trial site and assure that the monitor will receive appropriate support in their activities at the trial site, as agreed in separate contracts with each trial site. The declaration of informed consent (see Section 5.4) includes a statement to the effect that the monitor has the right – while observing the provisions of data protection legislation – to compare the case report forms (CRFs) with the trial subject's medical records (doctor's notes, ECGs, laboratory printouts etc.). The investigator will secure access for the monitor to all necessary documentation for trial-related monitoring. The aims of the monitoring visits are as follows:

- To check the declarations of informed consent
- To monitor trial subject safety (occurrence and documentation/reporting of AEs and SAEs)
- To check the completeness and accuracy of entries on the CRFs
- To validate the entries on the CRFs against those in the source documents (source data verification, SDV),
- To perform drug accountability checks
- To evaluate the progress of the trial
- To evaluate compliance with the trial protocol
- To assess whether the trial is being performed according to GCP at the trial site
- To discuss with the investigator aspects of trial conduct and any deficiencies found

A monitoring visit report is prepared for each visit describing the progress of the clinical trial and any problems (e.g. refusal to give access to documentation).

The exact extent of the monitoring procedures is described in a separate monitoring manual.

4.8.2. Audits/Inspections

As part of quality assurance, the sponsor has the right to audit the trial sites and any other institutions involved in the trial. The aim of an audit is to verify the validity, accuracy and completeness of data, to establish the credibility of the clinical trial, and to check whether the trial subject's rights and trial subject safety are being maintained. The sponsor may assign these activities to persons otherwise not involved in the trial (auditors). These persons are allowed access to all trial documentation (especially the trial protocol, case report forms, trial subjects' medical records, drug accountability documentation, and trial-related correspondence).

The sponsor and all trial sites involved undertake to support auditors and inspections by the competent authorities at all times and to allow the persons charged with these duties access to the necessary original documentation.

All persons conducting audits undertake to keep all trial subject data and other trial data confidential.

4.9. Documentation

All data relevant to the trial are documented soon after measurement by the investigator responsible in the electronic case report form supplied. Entering data may be delegated to members of the trial team. The CRFs are signed by the investigator.

4.9.1. Data management

The IT infrastructure and data management staff will be supplied by the ZKS Cologne. The trial database will be developed and validated before data entry based on standard operating

procedures at the ZKS Cologne. The data management system is based on commercial trial software and stores the data in a database. All changes made to the data are documented in an audit trail. The trial software has a user and role concept that can be adjusted on a trial-specific basis. The database is integrated into a general IT infrastructure and safety concept with a firewall and backup system. The data are backed up daily. After completion and cleaning of data, the database is locked and the data exported for statistical analysis.

The data will be entered online at the trial sites via the Internet. Plausibility checks are run during data entry, thereby detecting many discrepancies immediately. The ZKS Cologne Data Management will conduct further checks for completeness and plausibility and will clarify any questions with the trial sites electronically via the trial software. These electronic queries have to be answered by the trial site without unreasonable delay. Further details will be specified in the data management manual.

4.9.2. Archiving

All eCRFs, informed consent forms and other important trial materials will be archived for at least 10 years in accordance with §13 Sec. 10 of the GCP Regulations. Trial subject identification lists at each trial site will be stored separately from trial documentation.

5. Ethical and regulatory aspects

5.1. Independent ethics committee

The clinical trial will not be started before approval of the competent ethics committee.

In each trial site, the clinical study will not be started before approval of the competent local ethics committee concerning the suitability of the trial site and the qualifications of the investigators.

5.2. Ethical basis for the clinical trial

The present trial protocol and any amendments were and will be prepared in accordance with the Declaration of Helsinki in the version of October 1996 (48th General Assembly of the World Medical Association, Somerset West, Republic of South Africa).

5.2.1. Legislation and guidelines used for preparation

The present clinical trial will be conducted in accordance with the published principles of the guidelines for Good Clinical Practice (ICH-GCP) and applicable legislation (especially the Federal Drug Law [AMG] and the GCP-V). These principles cover, amongst other aspects, ethics committee procedures, the obtaining of informed consent from trial subjects, adherence to the trial protocol, administrative documentation, documentation regarding the IMP, data collection, trial subjects' medical records (source documents), documentation and reporting of adverse events (AEs), preparation for inspections and audits, and the archiving of trial documentation. All investigators and other staff directly concerned with the study will be informed that domestic and foreign supervisory bodies, the competent federal authorities and authorised representatives of the sponsor have the right to review trial documentation and the trial subjects' medical records at any time.

5.3. Notification of the authorities, approval and registration

Before the start of the clinical trial, all necessary documentation will be submitted to the competent supreme federal authority for approval (Paul Ehrlich Institute, Paul-Ehrlich-Institut [PEI]). The state authorities in each federal state in which the trial will be conducted will also be notified.

Before the trial is started, it will be registered under Current Controlled Trials (www.controlled-trials.com) or another trial register approved by the World Health Organisation (WHO) (http://www.who.int/ictrp/en/).

5.4. Obtaining informed consent from trial subjects

Trial subjects may not be enrolled into the present trial unless they have consented to take part in the trial after having been informed verbally and in writing in comprehensible language of the nature, scope and possible consequences by a trial investigator. Together with the consent to take part in the trial, the trial subject must also agree to representatives of the sponsor (e.g. monitors or auditors) or the competent supervisory or federal authorities having access to the data recorded within the framework of the clinical trial. The trial subject will be informed of the potential benefit and possible side effects of the IMP, It must be clear to trial subjects that he or she can withdraw his or her consent at any time without giving reasons and without jeopardizing his / her further course of treatment.

The originally signed consent form is archived in the investigator site file. Trial subjects receive copies of the written information sheet, confirmation of insurance with conditions, and the signed informed consent form. A copy of the written information sheet and the signed informed consent form will be filed in the patient's record.

The patient information sheet and informed consent form are supplied as an~~in~~ Appendix.

The patient information sheet, informed consent form, all other documents handed out to the trial subject and any recruitment advertisements must be submitted for approval before use to the ethics committee. Part of the monitoring activities are to check that the most recent informed consent form was used before the trial subject was enrolled and that it was dated and signed by the trial subject himself or herself.

5.5. Insurance of trial subjects

All trial subjects enrolled are insured in accordance with § 40 AMG under the group insurance contract of Charité University Medicine with HDI Gerling Versicherung (insurance company). The headquarters, policy number and telephone and fax number will be included in the patient information sheet.

5.6. Data protection

The provisions of data protection legislation will be observed. It is assured by the sponsor that all investigational materials and data will be pseudonymised in accordance with data protection legislation before scientific processing.

Trial subjects will be informed that their pseudonymised data will be passed on in accordance with provisions for documentation and notification pursuant to § 12 and § 13 of the GCP Regulations to the recipients described there. Subjects who do not agree that the information may be passed on in this way will not be enrolled into the trial.

6. Statistical methods and sample size calculation

6.1. Statistical and analytical plan

This is a phase II therapeutic-exploratory clinical trial with 60 patients randomly assigned to two treatment arms. Though the primary analysis is done at increase significance level (i.e. 10% one-sided), the conclusions can be strengthened if the observed significance level is lower (e.g. than 5% one-sided or even two-sided). The essentials of the statistical analysis are outlined below. Further details will be layed down in the statistical analysis plan.

6.1.1. Trial populations

All analyses will be conducted on three trial populations:

The primary dataset for analysis is derived from the intention-to-treat (ITT) population. This dataset includes all trial subjects enrolled into the trial and randomised - no exclusions.

The secondary dataset for analysis is derived from the per-protocol (PP) population. This dataset includes all trial subjects who were treated according to protocol (i.e. they received at least 3 injections of ranibizumab or at least one laser treatment as planned) and reached/provided the endpoints of main interest (i.e. after 6 months of follow-up).

The tertiary dataset for analysis is the safety population. This population includes all trial subjects who received any ranibizumab or laser treatment.

6.1.2. Description of trial subject groups

Demographic variables (including minimisation variables) and baseline values of study endpoints will be summarised by treatment group. Differences in location will be assessed by statistical hypothesis tests (i.e. chisquare- or t-tests).

6.1.3. Primary target variable

The primary variable change from baseline over 6 months (area under curve / 6 months) will be analysed by analysis of covariance with baseline and minimisation variables as covariates (type II SS). Missing values are imputed by the last observed value (possibly baseline). Robustness of results to various imputation approaches (best case, worst case, multiple imputation and pooling) are investigated in sensitivity analyses. ~~MMRM analyses are to complement the findings.~~

~~Moreover, ~~These will also include (A)~~a corresponding~~ mixed models for repeated measures (MMRM) ~~analysis will be performed (~~assuming missing at random, MAR (Gueorguieva 2004) and (B) permutation tests (Kalish 1985; Scott 2002).~~). The robustness of results due to various imputation scenarios will be assessed by sensitivity analysis (worst/best case, multiple imputation).~~

6.1.4. Secondary target variables

Analyses of secondary variables / endpoints follow the same lines as that of the primary variable. Safety data are summarized (multple-way contingency tables) and listed.

6.1.5. Subgroup analyses

Subgroup analyses will be done by radiation dose to the macula and disc (<=40gy:>40gy), tumor location (anterior/equator/posterior, 50:30:20), modalities of irradiation (e.g. Ru-plaque or proton beam) and study centre (Berlin/Essen) (50:50).

6.1.6. Interim analysis

Not applicable

6.2. Sample size calculation

Assuming a within-group standard deviation of 8,5 letters (calculated based on Finger et al., 2010), a sample size of 27 patients per treatment arm is sufficient to detect a clinically

relevant difference of 5 letters in the primary variable at a *one-sided level of 10%* with 80% power (δ/σ≈0.6). Note, this is a phase II therapeutic-exploratory trial. Randomisation will be stratified by radiation dose, tumor location and study centre. In order to account for stratification and drop-out, 27/0.9=30 patients will be randomised per treatment arm using Pocock's minimisation method with random element (Pocock 1975). Thus, altogether, 60 patients will be included in the trial. Power for the secondary endpoints (macular thickness, capillary dropout) may be much higher.

7. Safety

7.1. Definitions of adverse events and adverse drug reactions

7.1.1. Adverse event

An adverse event (AE) is any untoward medical occurrence in a trial subject administered an IMP. There does not necessarily have to be a causal relationship with this treatment.

All events occurring after the first administration of the IMP until 30 days after the patient has stopped the study must be documented,

The following events are excepted from reporting obligations and not considered as (S)AE:

- Injuries or trauma unrelated to the eye
- Concomitant disease

Concomitant diseases

The deterioration of a preexisting illness is also an AE in the context of a clinical trial. The following, however, is not regarded as an AE: a preexisting disease that led to a planned treatment measure before the start of the clinical trial, e.g. admission to hospital as an inpatient. This should be made clear in the trial subject's medical records and should also be documented in the CRF (see Section 7.1.3).

Pregnancy

For reasons of drug safety, the occurrence of a pregnancy during the conduct of this trial is to be regarded as an AE.

The investigator will inform the ZKS without delay about any pregnancy that occurs during the trial at latest within 24 hours of being made aware of it. This will be documented on a separate report form, "Pregnancy Report Form Part I". After delivery a "Pregnancy Report Form Part II" has to be filled in and must be sent to ZKS by the investigator within 24 hours of

awareness of childbirth. ZKS will forward these informations to the coordinating principle investigator. The pregnant women will be asked to give separate informed consent for pregnancy follow up. For tracing health of the newborn baby after delivery both parents have to give an informed consent.

In case of pregnancy study medication has to be stopped immediately.

7.1.2. Adverse drug reaction

An adverse drug reaction (ADR) is any noxious and unintended response to an investigational medicinal product (IMP) related to any dose with at least a reasonably possible causal relationship with the IMP.

7.1.3. Serious adverse events and serious adverse reactions

A serious AE (SAE) or serious ADR (SADR) is any untoward medical occurrence that at any dose

1. Results in death,
2. Is life-threatening at the time of the event
3. Requires inpatient hospitalisation or prolongation of existing hospitalisation, except:

- Routine treatment or monitoring of the studied indication, not associated with any deterioration in condition (e.g. Cataract formation)
- Elective or pre-planned treatment for a pre-existing condition that is unrelated to the indication under study and has not worsened since the start of study drug
- Treatment on an emergency outpatient basis for an event not fulfilling any of the definitions of a SAE given above and not resulting in hospital admission
- Social reasons and respite care in the absence of any deterioration in the patient's general condition

4. Results in persistent or significant disability/incapacity
5. Is a congenital anomaly or birth defect (1.-4.: § 3(8) GCP Regulations)
6. In the opinion of the investigator, fulfils any other criteria similar to 1.–4.

Inpatient hospitalisation is defined as any stay in hospital on the part of a trial subject that includes at least one night (midnight to 06:00). Admission to hospital as an inpatient planned before the first admission of the IMP are not SAEs, but must be documented in the proper manner in the trial subject's medical records and CRF (see Section 7.1.1).

If an AE is classified as an SAE, this is documented on a separate SAE sheet in addition to the standard AE documentation. The authorities must be notified of SAEs by law (for procedure, see 7.3).

7.1.4. Unexpected adverse drug reaction

An unexpected ADR is an ADR which, the nature or severity of which is not consistent with the applicable product information available for the IMP. Expected ADRs are listed in the appropriate reference documents (For Lucentis: Information Sheet for Health Professionals,. SmPC or other scientific (Up to Date) product information documents, if applicable)

7.1.5. Suspected unexpected serious adverse reactions

A suspected unexpected serious adverse reaction (SUSAR) is an adverse event the nature or severity of which is not consistent with the product information available for the IMP, is regarded as serious, and has at least a possible causal relationship with the IMP.

7.1.6. Other possible trial-specific complications or risks

As the trial concerns patients with choroidal melanoma, there is a theoretical possibility of recurrent melanoma during the trial.

- If a choroidal melanoma is documented to expand after radiation treatment, therapy is considered to have failed. The following criteria have been extablished to define tumor

growth according to the COMS study (Hawkins BS. Collaborative ocular melanoma study randomized trial of I-125 brachytherapy. Clin Trials. 2011 Oct;8(5):661-73):

Tumor expansion is considered in two stages:

1. suspected growth

2. documented growth

criteria for each of the parameters defining the stages are:

Suspected growth:

Confirmation of either of the following changes is considered adequate to define “suspected growth”:

• A 15% increase in tumor height, as determined from standardized echography

• A 250-micron expansion of any tumor boundary, as judged from fundus photography

Follow-up examinations at more frequent intervals are required after suspected growth.

For proton beam irradiation suspected growth during the first year is to be expected due to edema in the target tissue. However, thereafter no further increase in tumor size should be present.

Documented growth:

Tumors first have been ovserved to have changes that fulfill the criteria for suspected growth. Confirmation of either of the following changes within one year after suspected growth if confirmed is considered to be documented expansion:

• An additional 15% increase in tumor height, as determined from standardized echography.

• A further 250-micron growth of any tumor boundary, as judged from fundus photography.

If either of these criteria is met, radiation therapy is considered to have failed, and the eye should be managed to the discretion of the ophthalmologist.

Patients with documented tumor growth will receive the appropriate treatment to ascertain tumor control (e.g. radiation or transpupillary thermo therapy).

7.2. Documentation and follow-up of adverse events

The sponsor ensures that all persons involved in the treatment of trial subjects are adequately informed of the responsibilities and actions required when AEs occur. Trial subjects will be asked at each visit whether they have experienced AEs or SAEs. AEs will be documented in the trial subject’s medical records and in the CRF.

For the procedure of SAE-reporting see section 7.3, and section 4.7.1.3 for safety analyses.

7.2.1. Documentation of adverse events and adverse drug reactions

All AEs occurring during the defined reporting period will be documented in the CRF including all information listed below. Exempted are those AEs explicitly mentioned in Section 7.1.1.

The AE is documented in the CRF including the following information:

- Date and time of onset and resolution
- Severity
- Causal relationship with IMP / study treatment
- Seriousness
- Interruption or withdrawal of study treatment and other measures taken

Regardless of whether a causal relationship between the AE and the IMP is suspected, trial subjects who develop adverse events must be monitored until all symptoms have been subsided, pathological laboratory values have returned to pre-event levels, a plausible explanation is found for the AE, the trial subject has died, or the study has been terminated for the trial subject concerned.

Recurrent episodes, complications, or progression of the initial SAE must be reported as follow-up to the original episode, regardless of when the event occurs. An SAE that is considered completely unrelated to a previously reported one should be reported separately as a new event.

Follow-up observations: A Follow-up report must be send to ZKS immediately after significant changes of the medical conditions or if the event has finished for any reasons or at the last once a year prior to a DSUR.

Preexisting diseases (before administration of the IMP) are not documented as adverse events but as concomitant diseases. New diseases and preexisting diseases that worsen during the trial are documented as AEs.

7.2.2. Severity of the adverse event

The investigator will classify the severity of AEs as follows:

- Mild: clinical symptoms or signs that are well tolerated
- Moderate: clinical symptoms or signs that are enough to impair everyday activities
- Severe: clinical symptoms or signs that markedly impair the trial subject and result in inability to work or go about everyday activities

The severity of each AE must be assessed by using the NCI-CTC criteria, version 4.0

- Grade 1 = mild
- Grade 2 = moderate
- Grade 3 = severe
- Grade 4 = life-threatening or disabling
- Grade 5 = death related to AE

7.2.3. Causal relationship between adverse event and investigational medicinal product

The investigator will assess the for every AE whether a causal relationship with the IMP can be assumed or not. The assessment includes consideration of the nature and type of reaction, the temporal relationship with the IMP, the clinical status of the trial subject, concomitant medication and other relevant clinical factors. If the event is considered due to lack of efficacy or as a symptom or sign of the underlying disorder, no causal relationship will be assumed.

The following definitions are used to assess the causal relationship between all AEs and the IMP (for documentation in CRF, see also Section 7.2.2) (WHO Causality Assessment of Suspected Adverse Reactions):

- Certain: A clinical event, including laboratory test abnormality, occurring in a plausible time relationship to drug administration, and which cannot be explained by concurrent disease or other drugs or chemicals. The response to withdrawal of the drug (dechallenge) should be clinically plausible. The event must be definitive pharmacologically or phenomenologically, using a satisfactory rechallenge procedure if necessary.
- Probable/likely: A clinical event, including laboratory test abnormality, with a reasonable time sequence to administration of the drug, unlikely to be attributed to concurrent disease or other drugs or chemicals, and which follows a clinically reasonable response on withdrawal (dechallenge). Rechallenge information is not required to fulfill this definition.
- Possible: A clinical event, including laboratory test abnormality, with a reasonable time sequence to administration of the drug, but which could also be explained by concurrent disease or other drugs or chemicals. Information on drug withdrawal may be lacking or unclear.
- Unlikely: A clinical event, including laboratory test abnormality, with a temporal relationship to drug administration which makes a causal relationship improbable, and in which other drugs, chemicals or underlying disease provide plausible explanations.

- Conditional/unclassified: A clinical event, including laboratory test abnormality, reported as an adverse reaction, about which more data is essential for a proper assessment or the additional data are under examination.

  Unassessable/unclassifiable: A report suggesting an adverse reaction which cannot be judged because information is insufficient or contradictory, and which cannot be supplemented or verified.

An ADR is suspected if the causal relationship is at least ‘possible’ or ‘conditional/unclassified’ or ‘unassessable/unclassifiable’. Events assessed as ‘unlikely’ are not suspected ADRs.

7.3. Reporting of serious adverse events, pregnancy and changes in risk-benefit assessment

Regardless of the assumed causal relationship, every SAE that occurs during a trial must be documented in the appropriate part of the CRF and on an SAE sheet sent to the sponsor.

Information about all SAEs (Lucentis and laser treatment) is collected and recorded on an Serious Adverse Event Report Form (for example SAE form, see appendix B). The investigator must assess the relationship to study drug or laser treatment, complete the SAE Report Form in English, and send the completed, signed form by fax within 24 hours to the ZKS. The original copy of the SAE Report Form and the fax confirmation sheet must be kept with the case report form documentation at the study site. Follow-up information is sent to the same person to whom the original SAE Report Form was sent, using a new SAE Report Form stating that this is a follow-up to the previously reported SAE and giving the date of the original report. The follow-up information should describe whether the event has resolved or continues, if and how it was treated, whether the blind was broken or not, and whether the patient continued or withdrew from study participation. Novartis Pharmaceuticals will be notified for SAEs.

SAE reporting is based on regulatory requirements of AMG, but additionally all SAEs with a causal relationship to laser treatment should be reported to the responsible Ethic Committee in accordance with MPG §23b and Declaration of Helsinki.

Pregnancies must also be documented on separate pregnancy forms and reported to the sponsor within the defined periods (see Section 7.3.1.).

7.3.1. Reports from the investigator to the sponsor

The investigator will inform the ZKS-Köln of the occurrence or receipt of knowledge of the occurrence of an SAE/pregnancy without delay, at the latest within 24 hours of being made aware of the SAE.

Cologne Centre for Clinical Trials (ZKS Köln)

Gleueler Strasse 269

50935 CologneGermany

Tel.: +49 221 478 88158

Fax:+49 221 478 7984

The investigator will also inform the sponsor without delay about any pregnancy that occurs during the trial, i.e. within 24 hours of being made aware of such. This will be documented on a separate pregnancy form. The pregnant trial subject will be asked to give separate informed consent for pregnancy follow up.

All cases of suspected SAEs are assessed by the sponsor or PCI with regard to seriousness (see Section 7.1.3), causality (see Section 7.2.3) and expectedness (see Section 7.1.4), regardless of the investigator's assessments.

7.3.2. Unblinding when treatment is blinded

There is no blinded treatment in this trial.

7.3.3. Notification of ethics committee and competent supreme federal authority

Every SUSAR that becomes known in a clinical trial will be reported by the sponsor or PCI to the competent supreme federal authority, the responsible ethics committee, all principle investigators involved in the study and Novartis Pharma..

A CIOMS-1 format shall be used for submitting expedited reports to the above mentioned partners.

Fatal and life-threatening SUSARs

The competent supreme federal authority and the ethics committee responsible must be informed by the sponsor or PCI of all fatal or life-threatening SUSARs. This must be done without delay, at the latest 7 calendar days after becoming aware of the minimum criteria for reporting. In all cases, attempts must be made to obtain further relevant information which must be supplied to the competent supreme federal authority and the ethics committee within a further 8 days. Furthermore, if a trial subject dies, this information must be passed on to the ethics committee responsible for the region in which the death occurred.

SUSARs that are not fatal or life-threatening

The competent supreme federal authority and the ethics committee responsible will be informed without delay by the sponsor or PCI of all SUSARs, at the latest within 15 calendar days of becoming aware of the minimum criteria for reporting. Further relevant details will be passed on as soon as possible.

If the information at the time of reporting is incomplete, further information to enable adequate assessment of the case will be requested from the reporter or other available sources.

7.3.4. Review and reporting of changes in the risk-benefit ratio

Without delay, and at the latest within 15 days of the decision for the need to do so, the sponsor or PCI will inform the competent supreme federal authority, the ethics committee

responsible and the competent authorities of all other member states of the EU or EEA where the trial is being conducted, of any events or factors that mean that the risk-benefit ratio of the IMP has to be reviewed. These consist of especially:

- Individual reports of expected serious ADRs with an unexpected outcome
- A clinically relevant increase in the rate of occurrence of expected SADRs
- SUSARs in trial subjects who have already completed the follow-up period of the clinical trial (”end-of-trial visit”)
- Factors emerging in connection with trial conduct or the development of the IMP that may affect the safety of persons concerned.

7.3.5. Informing the Data Monitoring Committee

Not applicable

7.3.6. Informing the investigators

The sponsor or PCI will inform all principal investigators of each site of all SUSARs including all relevant further information within the periods set by the supreme federal authority. The principal investigator is responsible for disclosure of this information at the site.

If new information becomes known that is different from the scientific information given to the investigator, all investigators will be informed.

7.3.7. Informing the marketing authorisation holder

Novartis Pharmaceuticals will be notified for SAEs. The sponsor or PCI will also inform the marketing authorization holder about all SUSARs including information reported to the competent supreme authority and ethics committee in accordance with contractual agreements.

7.4. Annual safety report of trial subjects

Once per year, the sponsor or PCI will supply a report on the safety of trial subjects with all available relevant information concerning patient safety during the reference period to the competent supreme federal authority and the competent authorities of all other member states of the EU or EEA where the trial is being conducted. This report will also be supplied to the responsible ethics committee.

The annual safety report will be compiled according to the corresponding ICH guideline E2F „Development Safety Update Report – DSUR“

The data lock point for the patient data to be included and analyzed is 30.06.2006/ the last day before the date of approval of the first clinical trial investigating Lucentis by Novartis Pharma.

The sponsor or PCI will supply the report within 60 days of one year after the reference date (data-lock point).

8. Use of trial findings and publication

8.1. Reports

8.1.1. Interim reports

Section 7.4 describes the requirements for annual reports on the safety of trial subjects.

8.1.2. Final report

The competent authority and ethics committee will be informed within 90 days that the trial has officially ended.

Within one year of the completion of the trial, the competent federal authority and the ethics committee will be supplied with a summary of the final report on the clinical trial containing the principle results.

8.2. Publication

It is planned to publish the trial results, in mutual agreement with the PCI, in a scientific journal and at German or international congresses. Publication of the results of the trial as a whole is intended. Any publication will take account of the 'Uniform requirements for manuscripts submitted to biomedical journals (International Committee of Medical Journal Editors' (ICMJE) [JAMA 1997;277:927-34]).

The trial will also be registered in a public register in accordance with the recommendations of the ICMJE (see also Section 5.3).

Any published data will observe data protection legislation covering the trial subject and investigators. Success rates or individual findings at individual trial sites are known only to the sponsor.

Publications or lectures on the findings of the present clinical trial either as a whole or at individual investigation sites must be approved by the sponsor in advance, and the sponsor reserves the right to review and comment on such documentation before publication.

By signing the contract to participate in this trial, the investigator declares that he or she agrees to submission of the results of this trial to national and international authorities for approval and surveillance purposes, and to the Federal Physicians Association, the Association of Statutory Health Fund Physicians and to statutory health fund organisations, if required. At the same time, the investigator agrees that his or her name, address, qualifications and details of his or her involvement in the clinical trial may be made known to these bodies.

The support by the ZKS is to be mentioned in any publication. ZKS staff will be included as coauthors as applicable and the Grant number oft the ZKS (01KN1106) is mentioned in an acknowledgement. A copy of all publications will be sent to the ZKS.

9. Amendments to the trial protocol

To ensure that comparable conditions are achieved as far as possible at individual trial sites and in the interests of a consistent and valid data analysis, changes to the provisions of this trial protocol are not planned. In exceptional cases, however, changes may be made to the trial protocol. Such changes can only be made if agreed by the sponsor, sponsor's representative, the PCI and biometrician, and all Authors of this trial protocol. Any changes to the trial procedures must be made in writing and must be documented with reasons and signed by all Authors of the original trial protocol.

Amendments made in accordance with § 10 Secs. 1 and 4 GCP Regulations that require approval are submitted to the ethics committee and the supreme federal authority and will not be implemented until approved. Exceptions to this are amendments made to avoid immediate dangers.

10. References

1. The European Agency for the Evaluation of Medicinal Product. Note for Guidance on Good Clinical Practice (CPMP/ICH/135/95).
2. The European Agency for the Evaluation of Medicinal Product. Note for Guidance Structure and Content of Clinical Study Reports (CPMP/ICH/137/95).
3. National Cancer Institute. Protocol Templates, Applications and Guidelines http://ctep.cancer.gov/guidelines/templates.html.
4. EMEA-Guideline On Data Monitoring Committees: EMEA/CHMP/EWP/5872/03 Corr.
5. The DAMOCLES Study Group. A proposed charter for clinical trial 2005 data monitoring committees: helping them do their job well. Lancet 2005; 365: 711-22.
6. Clinical trial registration: a statement from the International Committee of Medical Journal Editors. Accessed at http://www.icmje.org/clin_trial.pdf on 22 May 2007.
7. WHO. Causality Assessment of Suspected Adverse Reactions. http://www.who-umc.org/DynPage.aspx?id=22682
8. Audren F, Erginay A, Haouchine B, Benosman R, Conrath J, Bergmann JF, Gaudric A, Massin P. Intravitreal triamcinolone acetonide for diffuse diabetic macular oedema: 6-month results of a prospective controlled trial. Acta Ophthalmol Scand. 2006 Oct;84(5):624-30.
9. Bartlema YM, Oosterhuis JA, Journee-De Korver JG, Tjho-Heslinga RE, Keunen JE. Combined plaque radiotherapy and transpupillary thermotherapy in choroidal melanoma: 5 years' experience. Br J Ophthalmol. 2003 Nov; 87(11):1370-3.
10. Char DH, Castro JR. Helium ion therapy for choroidal melanoma. Arch Ophthalmol. 1982 Jun;100(6):935-8.
11. Char DH, Crawford JB, Castro JR, Woodruff KH. Failure of choroidal melanoma to respond to helium ion therapy. Arch Ophthalmol. 1983 Feb;101(2):236-41.
12. Char DH, Huhta K, Waldman F. DNA cell cycle studies in uveal melanoma. Am J Ophthalmol. 1989 Jan 15;107(1):65-72.

13. Chaudhuri PR, Austin DJ, Rosenthal AR. Treatment of radiation retinopathy. Br J Ophthalmol. 1981 Sep;65(9):623-5.

14. Chang TS, Bressler NM, Fine JT, Dolan CM, Ward J, Klesert TR; MARINA Study Group. Improved vision-related function after ranibizumab treatment of neovascular age-related macular degeneration: results of a randomised clinical trial. Arch Ophthalmol. 2007 Nov;125(11):1460-9.

15. Chen, Y. et al. Selection and analysis of an optimized anti-VEGF antibody: crystal structure of an affinity-matured Fab in complex with antigen. J. Mol. Biol. 293, 865–881 (1999).

16. Dunavoelgyi R, Zehetmayer M, Simader C, Schmidt-Erfurth U. Rapid improvement of radiation-induced neovascular glaucoma and exudative retinal detachment after a single intravitreal ranibizumab injection. Clin Experiment Ophthalmol 2007;35:878-880.

17. Edelman JL, Lutz D, Castro MR. Corticosteroids inhibit VEGF-induced vascular leakage in a rabbit model of blood-retinal and blood-aqueous barrier breakdown. Exp Eye Res. 2005 Feb; 80(2):249-58.

18. Ferrara N, Davis-Smyth T. The biology of vascular endothelial growth factor. Endocr Rev. 1997 Feb; 18(1):4-25.

19. Finger PT, Kurli M. Laser photocoagulation for radiation retinopathy after ophthalmic plaque radiation therapy. Br J Ophthalmol 2005, 89, 730-738.

20. Finger PT. Anti-VEGF bevacizumab (Avastin) for radiation optic neuropathy. Am J Ophthalmol 2007, 143, 335-338.

21. Finger PT, Chin KJ. Intravitreous ranibizumab (lucentis) for radiation maculopathy. Arch Ophthalmol. 2010;128(2):249-5.

22. Finger PT. Radiation retinopathy is treatable with anti-vascular endothelial growth factor bevacizumab (Avastin). Int J Radiat Oncol Biol Phys 2008;70:974-977.

23. Finger PT, Chin K. Anti-vascular endothelial growth factor bevacizumab (avastin) for radiation retinopathy. Arch Ophthalmol 2007;125:751-756.

24. Funatsu H, Yamashita H, Noma H, Mimura T, Nakamura S, Sakata K, Hori S. Aqueous humor levels of cytokines are related to vitreous levels and progression of diabetic retinopathy in diabetic patients. Graefes Arch Clin Exp Ophthalmol. 2005 Jan; 243(1):3-8. Epub 2004 Jul 17.
25. Gass JD. A fluorescein angiographic study of macular dysfunction secondary to retinal vascular disease. VI. X-ray irradiation, carotid artery occlusion, collagen vascular disease, and vitritis. Arch Ophthalmol. 1968 Nov;80(5):606-17.
26. Gaudreault, J. et al. Preclinical pharmacokinetics of ranibizumab (rhuFabV2) after a single intravitreal administration. Invest. Ophthalmol. Vis. Sci. 46, 726–733 (2005).
27. Goodman DF, Char DH, Crawford JB, Stone RD, Castro JR. Uveal melanoma necrosis after helium ion therapy. Am J Ophthalmol. 1986 Jun 15;101(6):643-5.
28. Gragoudas ES, Goitein M, Verhey L, Munzenreider J, Suit HD, Koehler A. Proton beam irradiation. An alternative to enucleation for intraocular melanomas. Ophthalmology. 1980 Jun;87(6):571-81.
29. Gueorguieva R, Krystal JH. Move over ANOVA: progress in analyzing repeated-measures data and its reflection in papers published in the Archives of General Psychiatry. Archives of general psychiatry. 2004; 61(3): 310-7.
30. Gupta A, Muecke JS. Treatment of radiation maculopathy with intravitreal injection of bevacizumab (Avastin). Retina 2008;28:964-968
31. Guyer DR, Mukai S, Egan KM, Seddon JM, Walsh SM, Gragoudas ES. Radiation maculopathy after proton beam irradiation for choroidal melanoma. Ophthalmology. 1992 Aug;99(8):1278-85.
32. Harris JR, Levene MB. Visual complications following irradiation for pituitary adenomas and craniopharyngiomas. Radiology. 1976 Jul;120(1):167-71.
33. Hawkins BS. Collaborative ocular melanoma study randomized trial of I-125 brachytherapy. Clin Trials. 2011 Oct;8(5):661-73.
34. Heier JS, Antoszyk AN, Pavan PR, Leff SR, Rosenfeld PJ, Ciulla TA. Ranibizumab for treatment of neovascular age-related macular degeneration: a phase I/II multicenter, controlled, multidose study. Ophthalmology. 2006 Apr; 113(4):642.e1-4.

35. Höcht S, Bechrakis NE, Nausner M, Kreusel KM, Kluge H, Heese J, Heufelder J,Cordini D, Homeyer H, Fuchs H, Martus P, Foerster MH, Wiegel T, Hinkelbein W. Proton therapy of uveal melanomas in Berlin. 5 years of experience at the Hahn-Meitner Institute. Strahlenther Onkol. 2004;180(7):419-24.

36. Jaissle GB, Szurman P, Bartz-Schmidt KU. Ocular side effects and complications of intravitreal triamcinolone acetonide injection. Ophthalmologe. 2004 Feb; 101(2):121-8.

37. Kalish LA, Begg CB. Treatment allocation methods in clinical trials: A review. Statistics in Medicine. 1985; 4(2): 129-44.

38. Kijlstra A, La Heij E, Hendrikse F. Immunological factors in the pathogenesis and treatment of age-related macular degeneration. Ocul Immunol Inflamm. 2005 Feb; 13(1):3-11.

39. Mason JO, 3rd, Albert MA, Jr., Persaud TO, Vail RS. Intravitreal bevacizumab treatment for radiation macular edema after plaque radiotherapy for choroidal melanoma. Retina 2007;27:903-907.

40. Michels S, Rosenfeld PJ, Puliafito CA, Marcus EN, Venkatraman AS. Systemic bevacizumab (Lucentis®) therapy for neovascular age-related macular degeneration twelve-week results of an uncontrolled open-label clinical study. Ophthalmology. 2005 Jun; 112(6):1035-47.

41. Missotten GS, Notting IC, Schlingemann RO, Zijlmans HJ, Lau C, Eilers PH, Keunen JE, Jager MJ. Vascular endothelial growth factor a in eyes with uveal melanoma. Arch Ophthalmol. 2006 Oct; 124(10):1428-34.

42. Mitchell P, Bandello F, Schmidt-Erfurth U, Lang GE, Massin P, Schlingemann RO, Sutter F, Simader C, Burian G, Gerstner O, Weichselberger A; RESTORE study group. The RESTORE study: ranibizumab monotherapy or combined with laser versus laser monotherapy for diabetic macular edema. Ophthalmology. 2011 Apr;118(4):615-25.

43. Nauck M, Roth M, Tamm M, Eickelberg O, Wieland H, Stulz P, Perruchoud AP. Induction of vascular endothelial growth factor by platelet-activating factor and

platelet-derived growth factor is downregulated by corticosteroids. Am J Respir Cell Mol Biol. 1997 Apr; 16(4):398-406.

44. Nauck M, Karakiulakis G, Perruchoud AP, Papakonstantinou E, Roth M. Corticosteroids inhibit the expression of the vascular endothelial growth factor gene in human vascular smooth muscle cells. Eur J Pharmacol. 1998 Jan 12; 341(2-3):309-15.

45. Neufeld G, Cohen T, Gengrinovitch S, Poltorak Z. Vascular endothelial growth factor (VEGF) and its receptors. FASEB J. 1999 Jan; 13(1):9-22.

46. Oosterhuis JA, Journee-de Korver HG, Keunen JE. Transpupillary thermotherapy: results in 50 patients with choroidal melanoma. Arch Ophthalmol. 1998 Feb; 116(2):157-62.

47. Parsons JT, Bva FJ, Fitzgerald CR, Mendenhall WM, Million RR. Radiation retinopathy after external-beam irradiation: analysis of time-dose factors. Int J Radiat Oncol Biol Phys. 1994, 30, 765-773.

48. Pocock SJ, Simon R. Sequential treatment assignment with balancing for prognostic factors in the controlled clinical trial. Biometrics. 1975; 31(1): 103-15.

49. Rosenfeld PJ, Rich RM, Lalwani GA. Ranibizumab: Phase III clinical trial results. Ophthalmol Clin North Am. 2006 Sep; 19(3):361-72.

50. Scott NW, McPherson GC, Ramsay CR, Campbell MK. The method of minimization for allocation to clinical trials: a review. Control Clin Trials. 2002; 23(6): 662-74.

51. Shields CL, Demirci H, Dai V, Marr BP, Mashayekhi A, Materin MA, Manquez ME, Shields JA. Intravitreal triamcinolone acetonide for radiation maculopathy after plaque radiotherapy for choroidal melanoma. Retina. 2005 Oct-Nov; 25(7):868-74.

52. Shields CL, Naseripour M, Cater J, Shields JA, Demirci H, Youseff A, Freire J. Plaque radiotherapy for large posterior uveal melanomas (> or =8-mm thick) in 354 consecutive patients. Ophthalmology. 2002 Oct; 109(10):1838-49.

53. Van der Lelij A, Rothova A. Diagnostic anterior chamber paracentesis in uveitis: a safe procedure? Br J Ophthalmol. 1997 Nov; 81(11):976-9.

54. Wen JC, McCannel TA. Treatment of radiation retinopathy following plaque brachytherapy for choroidal melanoma. Curr Opin Ophthalmol. 2009 May;20(3):200-4.
55. Wilkes S, Kurland L, Campbell RJ, Robertson DM. Incidence of uveal malignant melanoma. Am J Ophthalmol. 1979 Sep;88(3 Pt 2):629-30.
56. Wimer BM. Curative potential of foremost mitogen applications. Cancer Biother Radiopharm. 2003 Dec; 18(6):903-16.
57. Ziemssen F, Voelker M, Altpeter E, Bartz-Schmidt KU, Gelisken F. Intravitreal bevacizumab treatment of radiation maculopathy due to brachytherapy in choroidal melanoma. Acta Ophthalmol Scand 2007;85:579-580.

11. Appendices

11.1. Trial sites and principle investigators
