# Supplementary material for: Influence of Ranibizumab versus laser photocoagulation on radiation retinopathy (RadiRet) - a prospective randomized controlled trial
Source: Graefes Arch Clin Exp Ophthalmol. 2020 Feb 28;258(4):869–78. doi: 10.1007/s00417-020-04618-7 (PMC7575494; doi:10.1007/s00417-020-04618-7)
Supplement: Supplementary file 2 — (DOCX 19 kb) [file 417_2020_4618_MOESM2_ESM.docx]

**Supplement**

Supplemental Table x: Subgroup analyses

|  |  | Mean±SD, n | Mean±SD, n | Mean±SD, n | ANCOVA (LOCF), EMM, 95% CI | |
| --- | --- | --- | --- | --- | --- | --- |
|  | Arm | Baseline | 26 weeks | 52 weeks | Average change (AUC)  over 26 weeks | Average change (AUC)  over 52 weeks |
| **BCVA (logMAR)** | |  |  |  |  |  |
| Dose to macula and disc | |  |  |  | 0.625 (interaction) | 0.212 (interaction) |
| ≤40 Gy | Ranibizumab | 0.50±0.24, 10 | 0.35±0.26, 9 | 0.70±0.42, 8 | -0.17, -0.28 to -0.05 | -0.10, -0.23 to 0.04 |
|  | Laser | 0.68±0.46, 10 | 0.60±0.49, 9 | 0.71±0.55, 7 | -0.06, -0.17 to 0.06 | -0.08, -0.21 to 0.06 |
|  | Contrast, 95% CI, p |  |  |  | 0.11, -0.06 to 0.27, 0.182 | 0.02, -0.17 to 0.21, 0.823 |
| >40 Gy | Ranibizumab | 0.79±0.36, 5 | 0.46±0.50, 4 | 0.89±0.81, 3 | -0.18, -0.34 to -0.02 | -0.16, -0.47 to 0.14 |
|  | Laser | 0.78±0.47, 6 | 1.06±0.45, 4 | 1.01±0.21, 4 | -0.01, -0.16 to 0.14 | 0.09, -0.19 to 0.37 |
|  | Contrast, 95% CI, p |  |  |  | 0.17, -0.05 to 0.39, 0.110 | 0.26, -0.16 to 0.67, 0.193 |
| **Central foveal thickness** | |  |  |  |  |  |
| Dose to macula and disc | |  |  |  | 0.233 (interaction) | 0.697 (interaction) |
| ≤40 Gy | Ranibizumab | 464±164, 10 | 397±143, 9 | 552±193, 8 | -75.9, -109.6 to -42.2 | -44.1, -83.0 to -5.3 |
|  | Laser | 508±134, 10 | 499±149, 9 | 463±146, 7 | -1.7, -35.4 to 32.0 | -5.9, -44.8 to 32.9 |
|  | Contrast, 95% CI, p |  |  |  | 74.2, 26.3 to 122.1, 0.005 | 38.2, -17.1 to 93.4, 0.163 |
| >40 Gy | Ranibizumab | 507±141, 5 | 365±172, 4 | 339±46, 2 | -60.3, -177.9 to 57.3 | -73.4, -217.6 to 70.9 |
|  | Laser | 512±185, 6 | 310±106, 3 | 454±254, 4 | -53.6, -161.0 to 53.7 | -57.5, -189.2 to 74.1 |
|  | Contrast, 95% CI, p |  |  |  | 6.7, -152.6 to 165.9, 0.925 | 15.8, -179.5 to 211.1, 0.857 |
| Abbr.: ANCOVA ‘Analysis of Covariance’ AUC ‘Area under curve’ BCVA ‘Best corrected visual acuity’ EMM ‘Estimated marginal mean', SD ‘Standard deviation’ LOCF ‘Last observation carried forward’ | | | | | | |
